# Supplementary material for: Closed-loop oxygen control for critically ill patients––A systematic review and meta-analysis
Source: PLoS One. 2024 Jun 12;19(6):e0304745. doi: 10.1371/journal.pone.0304745 (PMC11168613; doi:10.1371/journal.pone.0304745)
Supplement: S1 File — (DOCX) [file pone.0304745.s001.docx]

**Closed-loop Oxygen Control for Critically ill Patients––A Systematic Review and Meta-analysis**

Supporting information

**Supporting information figures**

**S1 Fig.** Summary of peripheral capillary oxygen saturation target definition of included studies.

**S2 Fig.** Forest plot of subgroup analysis of duration of intervention for the percentage of time spent in the SpO2 target

**S3 Fig.** Forest plot of subgroup analysis of patient’s condition (medical or post-surgical) for the percentage of time spent in the SpO2 target

**S4. Fig.** Sensitivity analyses for % of time spent in the SpO2 target

**S5. Fig.** Funnel plot for % of time spent in the SpO2 target

**S6 Fig.** Forest plot of percentage of time spent in hypoxemia

**S7 Fig.** Forest plot of percentage of time spent in hyperoxemia

**S8 Fig.** Sensitivity analysis for healthcare professionals' workload

**Supporting information tables**

**S1 Table.** Detailed search strategy for MEDLINE database

**S2 Table.** Detailed search strategy for CENTRAL database

**S3 Table.** Detailed search strategy for EMBASE database

**S4 Table.** Detailed search strategy for LILACS database

**S5 Table.** Detailed search strategy for LOVE evidence database

**S6 Table.** Detailed search strategy for CINAHL database

**S7 Table.** Awaiting classification studies

**S8 Table.** Ongoing studies

**S9 Table.** Clinical characteristics of included participants

**S10 Table.** Funding and sponsorship

**S11 Table.** Summary of the findings

**S12 Table.** Quantitative and qualitative data of adverse events of the included trials

**Supporting information references**

**e-References**

**S1 Fig.** Summary of peripheral capillary oxygen saturation target definition of included studies.


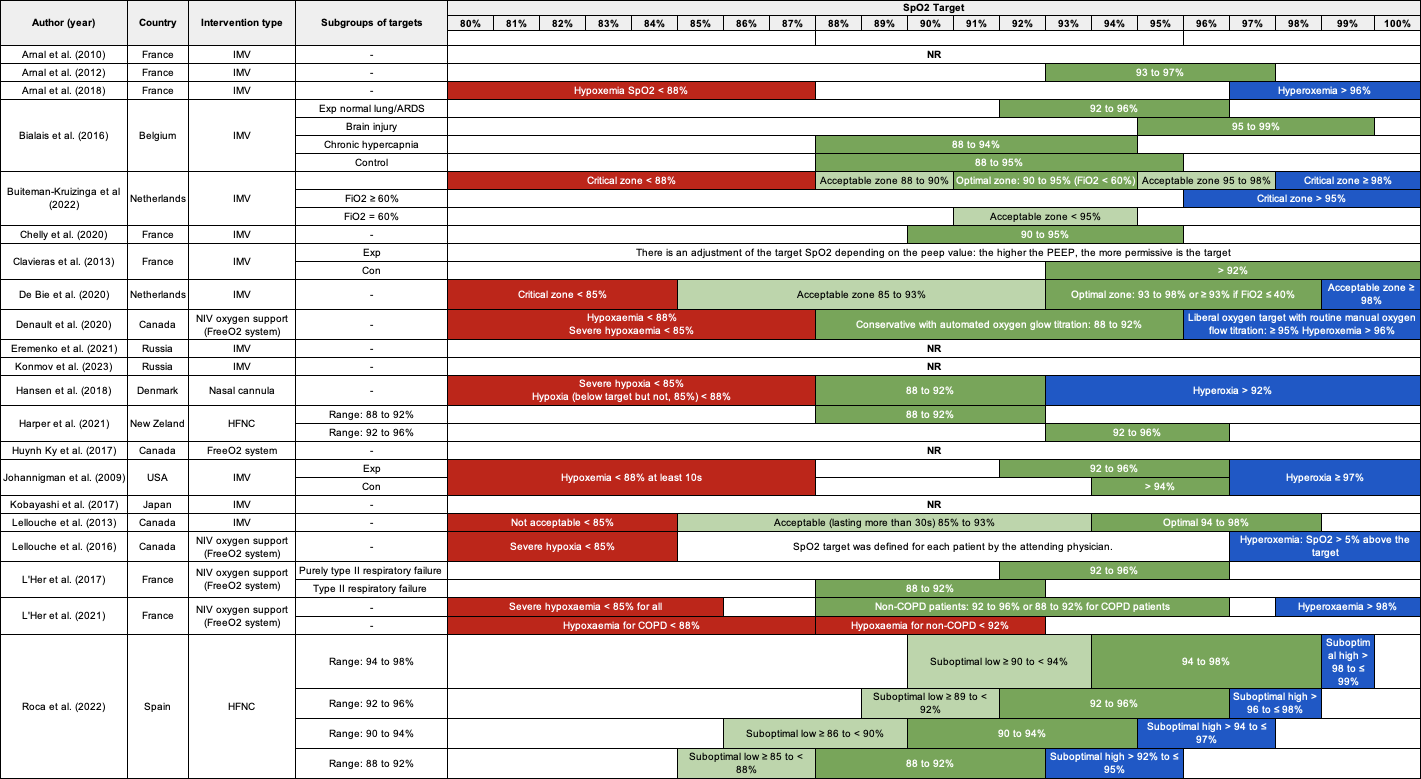


Color legends: blue = hyperoxia, hyperoxaemia or suboptimal high target; green = optimal saturation target; light green = suboptimal saturation target; red = hypoxia, hypoxaemia or severe hypoxaemia.

*Abbreviations:* ARDS = acute respiratory distress syndrome; Con = control; COPD = chronic obstructive pulmonary disease; Exp = experimental; FiO_2_ = fraction of inspired oxygen; HFNC = high flow nasal cannula; IMV = invasive mechanical ventilation; NIV = noninvasive ventilation; NR = not reported; PEEP = positive end-expiratory pressure; S = seconds; SpO_2_ = peripheral capillary oxygen saturation; USA = United States of America.

**S2 Fig.** Forest plot of subgroup analysis of duration of intervention for the percentage of time spent in the SpO2 target

**
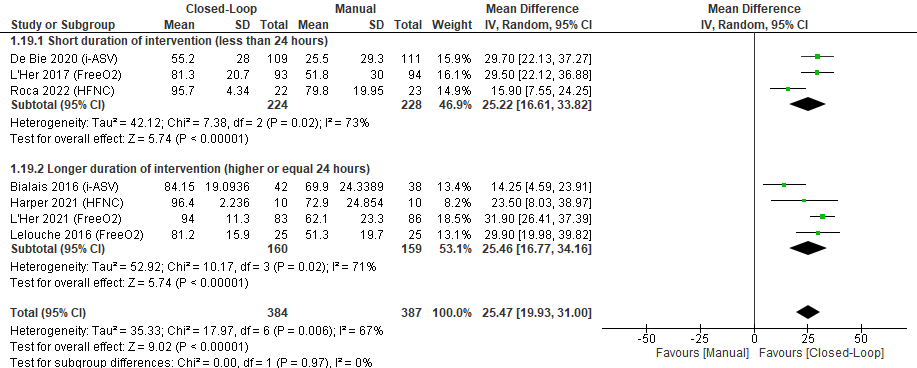
**

**S3 Fig.** Forest plot of subgroup analysis of patient’s condition (medical or post-surgical) for the percentage of time spent in the SpO2 target

**
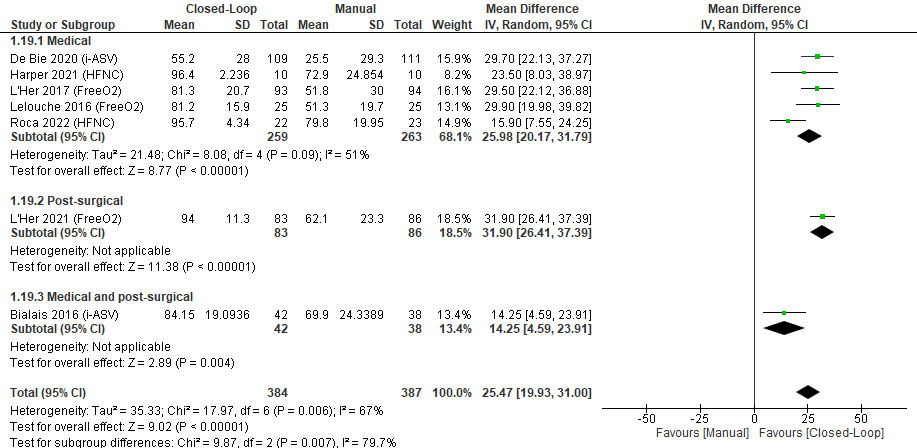
**

**S4. Fig.** Sensitivity analyses for the percentage of time spent in the SpO2 target


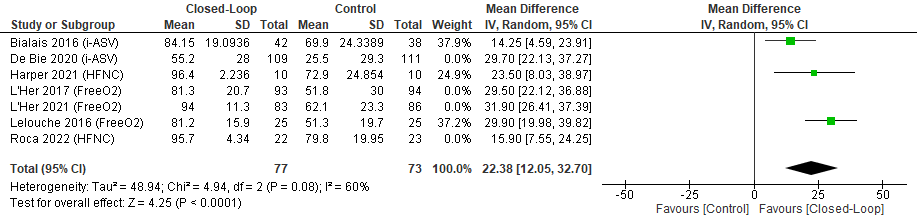


**S5. Fig.** Funnel plot for the percentage of time spent in the SpO2 target


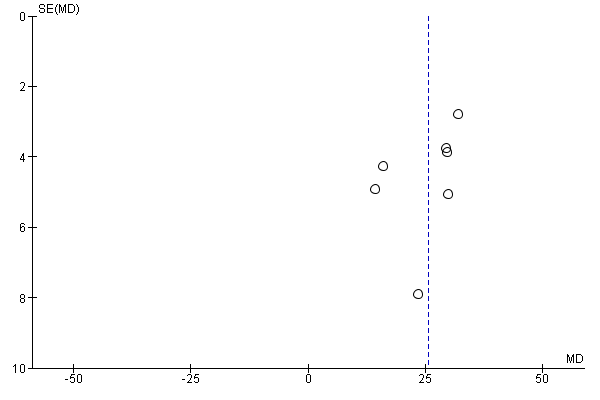


**S6 Fig.** Forest plot of percentage of time spent in hypoxemia


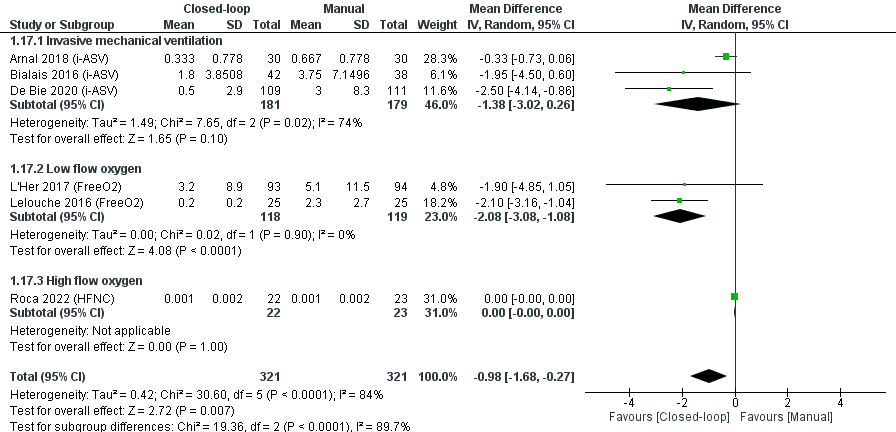


**S7 Fig.** Forest plot of percentage of time spent in hyperoxemia


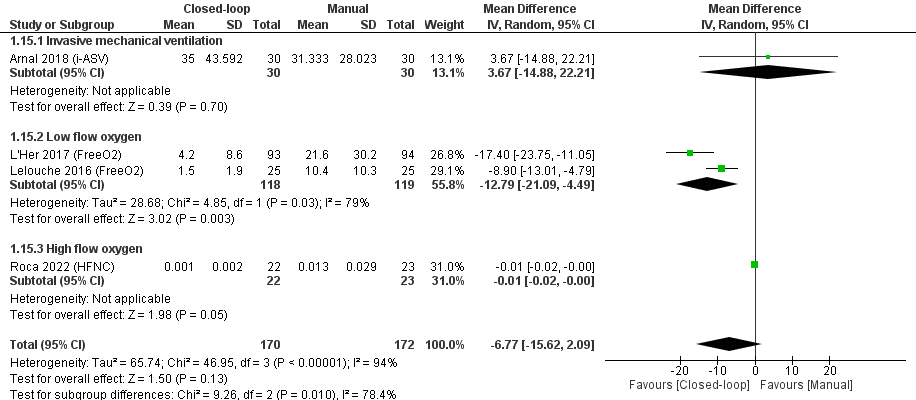


**S8 Fig.** Sensitivity analysis for healthcare professionals' workload


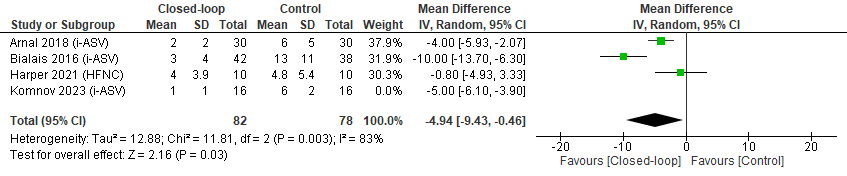


**S1 Table.** Detailed search strategy for MEDLINE database

| **Search strategy for MEDLINE** | |
| --- | --- |
| **Number** | **Search terms** |
| #1 | "Oxygen Inhalation Therapy"[Mesh] OR "Oxygen"[Mesh] OR oxygen*[tiab] OR Dioxygen[tiab] OR O2[tiab] OR FiO2[tiab] |
| #2 | concentrat*[tiab] OR inspir*[tiab] OR inhal*[tiab] OR level*[tiab] OR tension*[tiab] OR fraction*[tiab] OR arterial*[tiab] OR saturation supply*[tiab] OR supplement*[tiab] OR supplie*[tiab] OR therap*[tiab] OR administr*[tiab] OR dosag*[tiab] OR dose*[tiab] OR dosing*[tiab] OR titrat*[tiab] OR deliver*[tiab] |
| #3 | automat*[tiab] OR algorithms[tiab] OR system*[tiab] OR closed-loop[tiab] OR closed loop[tiab] OR intelligen*[tiab] OR targeted[tiab] OR machine learning[tiab] |
| #4 | adul*[All Fields] OR middle aged[sb] OR age[tw] OR aged[tiab] OR aged[MESH] OR geriatric*[tiab] OR geriatrics[MESH] OR elder*[tiab] OR olding[tiab] OR ageing[tiab] OR aging[tiab] OR aging[MESH] OR "frail elderly"[MESH]) |
| #5 | SpO2[tw] OR oxygen saturation[tw] OR Blood Oxygen Level*[tw] OR Saturation of Peripheral Oxygen[tw] OR oxygen weaning[tw] OR FiO2 weaning[tw] OR Length of stay*[tw] OR Cost*[tw] OR Adverse event*[tw] OR adverse effect*[tw] OR Near Misse*[tw] OR Side Effect*[tw] OR Adverse Reaction*[tw] OR Toxicity[tw] OR Mortalit*[tw] OR Fatality Rate*[tw] OR Death[tw] OR Workload*[tw] OR Work Load*[tw] OR process optimization[tw] OR Quality Improvement*[tw] |
| #6 | ((clinical[Title/Abstract] AND trial[Title/Abstract]) OR clinical trials as topic[MeSH Terms] OR clinical trial[Publication Type] OR random*[Title/Abstract] OR random allocation[MeSH Terms] OR therapeutic use[MeSH Subheading]) |
| #7 | #1 AND #2 AND #3 AND #4 AND #5 AND #6 |
| #8 | (animals [mh] NOT humans [mh]) |
| #9 | #7 NOT #8 |

*Abbreviation:* MEDLINE = medical literature analysis and the retrieval system online

**S2 Table.** Detailed search strategy for CENTRAL database

| **Search strategy for CENTRAL** | |
| --- | --- |
| **Number** | **Search terms** |
| #1 | [mh "Oxygen Inhalation Therapy"] OR [mh Oxygen] OR (oxygen* OR Dioxygen OR O2 OR FiO2):ti,ab |
| #2 | (concentrat* OR inspir* OR inhal* OR level* OR tension* OR fraction* OR arterial* OR saturation NEXT supply* OR supplement* OR supplie* OR therap* OR administr* OR dosag* OR dose* OR dosing* OR titrat* OR deliver*):ti,ab |
| #3 | (automat* OR algorithms OR system* OR closed-loop OR (closed NEXT loop) OR intelligen* OR targeted OR machine NEXT learning):ti,ab |
| #4 | adul* OR middle aged OR age |
| #5 | (aged OR geriatric* OR elder* OR olding OR ageing OR aging):ti,ab |
| #6 | [mh Aged] OR [mh geriatrics] OR [mh aging] OR [mh "frail elderly"] |
| #7 | {OR #4-#6} |
| #8 | SpO2 OR (oxygen saturation) OR ("Blood Oxygen" NEXT Level*) OR (Saturation of Peripheral Oxygen) OR (oxygen weaning) OR (FiO2 weaning) OR ("Length of" NEXT stay*) OR Cost* OR ("Adverse" NEXT event*) OR ("adverse" NEXT effect*) OR ("Near" NEXT Misse*) OR ("Side" NEXT Effect*) OR ("Adverse" NEXT Reaction*) OR Toxicity OR Mortalit* OR ("Fatality" NEXT Rate*) OR Death OR Workload* OR ("Work" NEXT Load*) OR (process optimization) OR ("Quality" NEXT Improvement*)  #9 |
| #9 | #1 AND #2 AND #3 AND #7 AND #8 |

*Abbreviation:* CENTRAL = cochrane central register of controlled trials.

**S3 Table.** Detailed search strategy for EMBASE database

| **Search strategy for EMBASE** | |
| --- | --- |
| **Number** | **Search terms** |
| #1 | 'oxygen therapy'/exp OR 'oxygen'/exp OR oxygen*:ab,ti OR Dioxygen:ab,ti OR O2:ab,ti OR FiO2:ab,ti |
| #2 | concentrat*:ab,ti OR inspir*:ab,ti OR inhal*:ab,ti OR level*:ab,ti OR tension*:ab,ti OR fraction*:ab,ti OR arterial*:ab,ti OR 'saturation supply*':ab,ti OR supplement*:ab,ti OR supplie*:ab,ti OR therap*:ab,ti OR administr*:ab,ti OR dosag*:ab,ti OR dose*:ab,ti OR dosing*:ab,ti OR titrat*:ab,ti OR deliver*:ab,ti |
| #3 | #3 automat*:ab,ti OR algorithms:ab,ti OR system*:ab,ti OR 'closed-loop':ab,ti OR 'closed loop':ab,ti OR intelligen*:ab,ti OR targeted:ab,ti OR 'machine learning':ab,ti |
| #4 | adul*/mj OR 'middle aged'/de OR age/br OR 'aged'/de OR 'frail elderly'/de OR 'geriatrics'/de OR 'very elderly'/de OR 'aging'/de OR geriatric*:ti,ab OR elder*:ti,ab OR old*:ti,ab OR ageing:ti,ab OR aging:ti,ab |
| #5 | SpO2/br OR 'oxygen saturation'/br OR 'Blood Oxygen Level*'/br OR 'Saturation of Peripheral Oxygen'/br OR 'oxygen weaning'/br OR 'FiO2 weaning'/br OR 'Length of stay*'/br OR Cost*/br OR 'Adverse event*'/br OR 'adverse effect*'/br OR 'Near Misse*'/br OR 'Side Effect*'/br OR 'Adverse Reaction*'/br OR Toxicity/br OR Mortalit*/br OR 'Fatality Rate*'/br OR Death/br OR Workload*/br OR 'Work Load*'/br OR 'process optimization'/br OR 'Quality Improvement*'/br  #6 |
| #6 | 'crossover procedure':de OR 'double-blind procedure':de OR 'randomized controlled trial':de OR 'single-blind procedure':de OR (random* OR factorial* OR crossover* OR cross NEXT/1 over* OR placebo* OR doubl* NEAR/1 blind* OR singl* NEAR/1 blind* OR assign* OR allocat* OR volunteer*):de,ab,ti |
| #7 | #1 AND #2 AND #3 AND #4 AND #5 AND #6 |
| #8 | ([animals]/lim NOT [humans]/lim) |
| #9 | #7 NOT #8 |
| #10 | #9 AND [embase]/lim NOT ([embase]/lim AND [medline]/lim) |

*Abbreviation:* EMBASE = excerpta medica database.

**S4 Table.** Detailed search strategy for LILACS database

| **Search strategy for LILACS** | |
| --- | --- |
| **Number** | **Search terms** |
| #1 | MH:"Oxigenoterapia" OR MH:"Oxygen Inhalation Therapy" OR MH:"Terapia por Inhalación de Oxígeno" OR MH:E02.880.690$ OR MH:"Oxigênio" OR MH:"Oxygen " OR MH:"Oxígeno" OR MH:D01.268.185.550$ OR MH:D01.362.670$ OR MH:SP4.097.063.949$ OR TW:oxygen* OR TW:Dioxygen OR TW:O2 OR TW:FiO2 |
| #2 | TW:concentrat* OR TW:inspir* OR TW:inhal* OR TW:level* OR TW:tension* OR TW:fraction* OR TW:arterial* OR (TW:saturation supply*) OR TW:supplement* OR TW:supplie* OR TW:therap* OR TW:administr* OR TW:dosag* OR TW:dose* OR TW:dosing* OR TW:titrat* OR TW:deliver* |
| #3 | TW:automat* OR TW:algorithms OR TW:system* OR TW:closed-loop OR (TW:closed loop) OR TW:intelligen* OR TW:targeted OR (TW:machine learning) |
| #4 | TW:adul* OR MJ:"middle aged" OR TW:age OR TW:aged OR TW:aged OR TW:geriatric* OR MH:"geriatrics" OR TW:elder* OR TW:olding OR TW:ageing OR TW:aging OR MH:"aging" OR MH:"frail elderly" |
| #5 | TW:SpO2 OR (TW:oxygen saturation) OR (TW:Blood Oxygen Level*) OR (TW:Saturation of Peripheral Oxygen) OR (TW:oxygen weaning) OR (TW:FiO2 weaning) OR (TW:Length of stay*) OR TW:Cost* OR (TW:Adverse event*) OR (TW:adverse effect*) OR (TW:Near Misse*) OR (TW:Side Effect*) OR (TW:Adverse Reaction*) OR TW:Toxicity OR TW:Mortalit* OR (TW:Fatality Rate*) OR TW:Death OR TW:Workload* OR (TW:Work Load*) OR (TW:process optimization) OR (TW:Quality Improvement*) |
| #6 | TW:clinical AND TW:trial OR MH:"clinical trials" OR PT:"clinical trial" OR TW:random* OR MH:"random allocation" OR SH:"therapeutic use" |
| #7 | #1 AND #2 AND #3 AND #4 AND #5 AND #6 |

*Abbreviation:* LILACS = latin american the caribbean literature in health sciences.

**S5 Table.** Detailed search strategy for LOVE evidence database

| **Search strategy for LOVE evidence** | |
| --- | --- |
| **Number** | **Search terms** |
| #1 | (Oxygen Inhalation Therapy) OR Oxygen OR oxygen* OR Dioxygen OR O2 OR FiO2 |
| #2 | concentrat* OR inspir* OR inhal* OR level* OR tension* OR fraction* OR arterial* OR "saturation supply*" OR supplement* OR supplie* OR therap* OR administr* OR dosag* OR dose* OR dosing* OR titrat* OR deliver* |
| #3 | automat* OR algorithms OR system* OR closed-loop OR intelligen* OR targeted OR (machine learning) |
| #4 | #1 AND #2 AND #3 |

**S6 Table.** Detailed search strategy for CINAHL database

| **Search strategy for CINAHL** | |
| --- | --- |
| **Number** | **Search terms** |
| #1 | (MM "Oxygen Therapy+") OR (MM "Oxygen+") OR oxygen* OR Dioxygen OR O2 OR FiO2  AND |
| #2 | TX (concentrat* OR inspir* OR inhal* OR level* OR tension* OR fraction* OR arterial* OR (saturation supply*) OR supplement* OR supplie* OR therap* OR administr* OR dosag* OR dose* OR dosing* OR titrat* OR deliver*)  AND |
| #3 | TX (automat* OR algorithms OR system* OR closed-loop OR (closed loop) OR intelligen* OR targeted OR (machine learning))  AND |
| #4 | (MM "Aged+") OR (MM "Geriatrics") OR (MM "Aging+") OR (MM "Frail Elderly") OR TX (adul* OR middle aged OR age OR aged OR geriatric* OR elder* OR olding OR ageing OR aging)  AND |
| #5 | TX (SpO2 OR (oxygen saturation) OR (Blood Oxygen Level*) OR (Saturation of Peripheral Oxygen) OR (oxygen weaning) OR (FiO2 weaning) OR (Length of stay*) OR Cost* OR (Adverse event*) OR (adverse effect*) OR (Near Misse*) OR (Side Effect*) OR (Adverse Reaction*) OR Toxicity OR Mortalit* OR (Fatality Rate*) OR Death OR Workload* OR (Work Load*) OR (process optimization) OR (Quality Improvement*))  AND |
| #6 | TW (clinical AND trial) OR (MM "Clinical Trials+") OR (TW random*) OR (MM "Random Assignment") OR PT clinical trial |

*Abbreviation:* CINALH – cumulative index to nursing and allied health literature.

**S7 Table.** Awaiting classification studies

| **Author**  **(year)**  **[Country]** | **Participants** | **Interventions** | **Outcomes** | **Notes** |
| --- | --- | --- | --- | --- |
| Bialais et al.^18^  (2013)  [Belgium] | *n* = 24  - Exp, *n*: 13  - Con, *n*: 11 | - Exp: INTELLiVENT^®^-ASV ventilation mode - Con: conventional ventilation. | - Time spent by the various parameters in the suboptimal zone, time spent in the optimal zone, number of manual adjustments, ventilatory parameters, oxygenation parameters and blood gasses - Duration: 48 hours | - Trial published in the Meeting abstracts of 33rd International Symposium on Intensive Care and Emergency Medicine. - Awaiting response after contact via email. |
| Bialais et al.^19^  (2013)  [Belgium] | *n* = 80 | - Exp: INTELLiVENT^®^-ASV ventilation mode - Con: conventional ventilation | - Ventilation and oxygenation parameter, time spent in ventilation ranges was calculated for SpO_2_, EtCO_2_, Vt, and RR. - Duration: 48 hours | - Conference abstract published in the 26th ESICM Annual Congress (2013). - Awaiting the authors' response. We were uncertain about the possibility of being a report of a study that is already included in our review. |
| Malika et al.^20^  (2018)  [France] | *n* = 60  - Exp, *n*: 30  - Con, *n*: 30 | - Exp: INTELLiVENT^®^-ASV mode - Con: conventional ventilation | - Number of arterial blood gasses, percentage of time of ventilation spent with SpO_2_ > 96% and with SpO_2_ < 88% - Duration: NR | - Conference abstract published in the 26th ESICM Annual Congress. - We were uncertain about the possibility of being a report of a study that is already included in our review. |
| Ye et al.^21^  (2016)  [China] | *n* = NR | - Exp: INTELLiVENT^®^-ASV ventilation mode - Con: ASV mode. | - FiO_2_, PEEP, VE, SpO_2_, PEtCO_2_, Vt, RR and static lung compliance inspiratory pressure, vital parameters of signs hemodynamics and ABG. - Duration: 2 hours | - Part of a special issue of the 27th Great Wall International Congress of Cardiology. International Society of Cardiovascular Pharmacotherapy International Congress of Cardiovascular Prevention and Rehabilitation (2016). - Awaiting response after contact via email. |

*Abbreviations:* ABG = arterial blood gas; ASV = adaptive support ventilation; Con = control; ESICM = European society of intensive medicine; Exp = experimental; FiO_2_ = inspired fraction of oxygen; ICU = intensive care unit; VE = minute volume; NR = not reported; PEEP = positive end-expiratory pressure; PEtCO_2_ = end-tidal carbon dioxide tension; RR = respiratory rate; SpO_2_ = peripheral oxygen saturation; Vt = tidal volume.

**S8 Table.** Ongoing studies

|  | **Principal investigator** | **Trial registry** | **Country** | **Last update** | **Recruitment status** | **Methods** |
| --- | --- | --- | --- | --- | --- | --- |
| 1 | Andersen et al.  (2019) | NCT04079465 | Denmark | December 29, 2021 | Recruiting | - Exp = usual care + ‘O2matic’ controlled oxygen therapy for a maximum of 24 hours or until weaning from oxygen supplementation - Con = usual care + manual controlled oxygen therapy by nursing staff, ‘O2matic’ is used in monitoring mode to measure SpO_2_ continuously |
| 2 | Bech et al.  (2020) | NCT04375917 | Denmark | October 26, 2022 | Completed, not published | - Exp = usual care + ‘O2matic’ controlled oxygen therapy for a maximum of 3 days or until weaning from oxygen supplementation - Con = usual care + manual controlled oxygen therapy by nursing staff, ‘O2matic’ is used in monitoring mode to measure SpO_2_ continuously |
| 3 | Dekker et al.  (2022) | Netherlands Trial Register / Main ID: NTR6061 | The Netherlands | August 18, 2022 | Pending | - Exp = MV with INTELLiVENT^®^–ASV after surgery - Con = MV based on the conventional modes (e.g.: VCV or PCV, and PSV) |
| 4 | Hansen et al.  (2018) | NCT03661086 | Denmark | September 21, 2022 | Completed, not published | - Exp = usual care + ‘O2matic’ controlled oxygen therapy for a maximum of 3 days or until weaning from oxygen supplementation - Con = usual care plus manual controlled oxygen therapy by nursing staff. O2matic is used in monitoring mode to measure SpO_2_ continuously |
| 5 | Hôpital Foch* Responsible Party (2021) | NCT05009628 | France | January 19, 2022 | Completed, not published | - Exp = automated oxygenation with the patient lying down position - Con = control oxygenation with the patient in a sitting position |
| 6 | Horn et al.  (2020) | NCT04593810 | Netherlands and Italy | July 20, 2023 | Recruiting | - Exp = automated ventilation INTELLiVENT^®^-ASV - Con = conventional ventilation not fully automated (e.g.: VCV, PCV, and PSV) |
| 7 | Hove et al.  (2022) | NCT05452863 | Denmark | October 18, 2022 | Recruiting | - Exp = oxygen delivered by the ‘O2matic’ device - Con = oxygen delivered by nurse adjustments in the usual way |
| 8 | Jolliet et al.  (2012) | NCT01695603 | Switzerland | May 8, 2017 | Completed, not published | - Exp = brain injured patients ventilated during 2 hours with INTELLiVENT^®^-ASV automated mode - Con = brain injured patients ventilated during 2 hours with a standard mode |
| 9 | Kirton et al.  (2021) | ACTRN12621000658819 | New Zealand | June 6, 2023 | Completed, not published | - Exp = HFNC delivered by Airvo™ 3 device with automatic oxygen titration - Con = oxygen device requiring manual adjustment |
| 10 | Kirton et al.  (2022) | ACTRN12622000433707 | New Zealand | June 5, 2023 | Completed | - Exp = oxygen delivered by the Airvo™ 3 acute device using its closed loop oxygen control function - Con = manual oxygen control |
| 11 | Kirton et al.  (2022) | ACTRN12622000132741 | New Zealand | May 15, 2023 | Completed | - Exp = NIV therapy, including Bi-level S/T NIV and CPAP, using the Airvo™ 3 device with closed-loop oxygen titration - Con = oxygen device requiring manual adjustment |
| 12 | Lellouche et al.  (2013) | NCT03122210 | Canada | December 20, 2017 | Completed, not published | - Exp = automated adjustment of oxygen during patient hospitalization using ‘FreeO2’ device with a SpO2 target of 92% or 97% - Con: Manual adjustment of oxygen during patient hospitalization by hospital staff |
| 13 | Lellouche et al.  (2019) | NCT03835741 | Canada | April 4, 2023 | Recruiting | - Exp = automated adjustment of oxygen during patient hospitalization by ‘FreeO2’ device - Con: Manual adjustment of oxygen during patient hospitalization by hospital staff |
| 14 | Lellouche et al.  (2020) | NCT04320056 | Canada | April 21, 2020 | Unknown | - Exp = automated oxygen administration delivered with ‘FreeO2’ device - Con = oxygen delivered as per usual local practices |
| 15 | Lim et al  (2022) | KCT0008038 | Republic of Korea | July 12, 2023 | Completed, not published | - Exp = target SpO_2_ feedback system mode for HFNC - Con = conventional mode of HFNC |
| 16 | Noel-Savina et al.  (2018) | NCT03527992 | France | July 11, 2023 | Recruiting | - Exp = automated oxygen therapy intervention, where the oxygen administration is automatically adjusted to the patients’ saturation, which is continuously monitored - Con = oxygen standard therapy |
| 17 | Rittayamai et al  (2022) | TCTR20220801007 | Thailand | August 01, 2022 | Not yet recruiting | - Exp = high-velocity nasal insufflation with automate oxygen controller: T = 37°C, flow rate 50 L/min and automate oxygen controller with target SpO_2_ equal to 94% (92-98%) for 60 min - Con = conventional high-velocity nasal insufflation: T = 37°C, flow rate 50 L/min and FiO_2_ is adjusted to maintain SpO_2_ equal to or more than 94% |
| 18 | Yangmei et al.  (2022) | NCT05452759 | China | July 19, 2022 | Not yet recruiting | - Exp = new type of tracheotomy high-flow oxygen therapy (NTHF) + heated humidifier (MR850), T = 37°C automatically adjusted to maintain the gas temperature at the entrance of the tracheal tube, and SpO_2_ between 94-100% - Con = Airvo™ 2, select the output gas of T = 37°C, monitor and maintain SpO_2_ between 94-100%, and adjust the output gas flow rate of the therapy device to 40, 50, and 60L/min, respectively |

*Abbreviations:* ACTRN = Australian clinical trials registration number; C = celsius; Con = control; CPAP = continuous positive airway pressure; Exp = experimental; HFNC = high flow nasal cannula; ICU = intensive care unit; FiO_2_ = fraction of inspired oxygen; hr = hour; KCT = Korean clinical trial; L = liter; min = minute; NIV = noninvasive ventilation; NTHF = new artificial airway high flow humidification oxygen therapy device; PCV = pressure controlled ventilation; PSV = pressure support ventilation; S/T = spontaneous/timed; SpO_2_ = peripheral oxygen saturation; T = temperature; TCTR = Thai Clinical Trials Registry; VCV = volume controlled ventilation.

**S9 Table.** Clinical characteristics of included participants

| **Study**  **(year)**  **Country**  **[Study type]** | **Comorbidities** | **Diseases** | **Type of hypoxemia** | **Length of Stay / Mortality** | **Severity Scores** | |
| --- | --- | --- | --- | --- | --- | --- |
|  |  |  |  |  | **Experimental** | **Control** |
| Arnal et al.^10^ (2010)  France  [RCT crossover] | NR | - *n* = ARDS: 28, normal lungs: 15 | Acute hypoxemia | - LOS = NR - Mortality = NR | NR | |
| Arnal et al.^16^  (2012)  France [RCT crossover] | NR | - *n* (%) = coma, stroke, head trauma, meningitis: 12 (24), sepsis: 7 (14), ARDS caused by pulmonary injury (pneumonia, aspiration, chest trauma): 20 (40), ARDS caused by extra-pulmonary injury (septic shock, pancreatitis, transfusion-related acute lung injury): 11 (22) | Acute hypoxemia | - LOS = NR - Mortality = NR | - SAPS II, mean (SD) = 50 (18) | |
| Arnal et al.^22^  (2018) France  [RCT] | NR | - Exp, *n* (%) = ARDS: 8 (27), chest wall stiffness: 7 (23), COPD: 3 (10) and others: 3 (10). - Con, *n* (%) = ARDS: 10 (33), chest wall stiffness: 5 (17), COPD: 4 (13). | Acute hypoxemia | - ICU LOS, median [IQR] = Exp: 10 [6-13]; Con: 9 [5-14] - Hospital LOS, median [IQR] = Exp: 18 [12-29]; Con: 19 [13-32] - 28-day in ICU Mortality, *n* (%) = Exp: 9 (30); Con: 7 (23). | - SAPS II, mean (SD) = 53 (14) | - SAPS II, mean (SD) = 53 (14) |
| Bialais et al.^23^  (2016)  Belgium  [RCT] | - Chronic respiratory disease, chronic cardiac insufficiency, atrial fibrillation and obesity | - Exp = ARDS, post-surgery, pneumonia, brain injury, sepsis, polytraumatism, ADCPD and others*. - Con = ARDS, post-surgery, pneumonia, brain injury, sepsis, polytraumatism, ADCPD and others*. | Acute and Chronic exacerbated hypoxemia | - ICU LOS, median [95% CI] = Exp: 11.5 [10.8-20.8]; Con: 13.0 [11.6-24.3]. - Hospitalar LOS, median [95% CI] = Exp: 38 [34.5-75.3]; Con: 46.0 [41.0-72.7]. - Mortality, *n* (%) = Exp: 9 (21); Con: 11 (29). - Total mortality, *n* (%) = Exp: 10 (24); Con: 16 (42). | - APACHE II, median [IQR] = 23 [19-29] | - APACHE II, median [IQR] = 24 [17-29] |
| Buiteman-Kruizinga et al.^15^  (2022) Netherlands  [RCT crossover] | NR | NR | NR | - LOS = NR - Mortality = NR | NR | NR |
| Chelly et al.^11^  (2020)  France  [RCT crossover] | - *n* (%) = COPD: 55 (21), chronic respiratory failure: 26 (10), chronic heart failure: 34 (13) | - *n* (%) = ARF: 138 (52), coma: 67 (25), cardiac arrest: 19 (7), sepsis: 15 (6), other: 24 (9), unknown: 2 (1). | Acute hypoxemia | - LOS = NR - Mortality = NR | - SAPS II at ICU admission, mean (SD) = 59 (19), [95% CI, 56-61]  - SOFA at inclusion, mean (SD) = 9 (3), [95% CI, 8-9]. | |
| Clavieras et al.^12^  (2013)  France  [RCT crossover] | NR | - Exp, *n* = septic shock–pneumonia: 3, liver transplant: 2, septic shock peritonitis: 2, liver traumatism: 1, pneumonia: 1, peritonitis: 3, hepatic encephalitis: 1, polytraumatized: 1 - Con, *n* = septic shock–pneumonia: 3, liver transplant: 2, septic shock–peritonitis: 2, liver traumatism: 1, pneumonia: 1, peritonitis: 3, hepatic encephalitis: 1, polytraumatized: 1 | Acute hypoxemia | - LOS = NR - Mortality = NR | - SAPS II, median [IQR] = 38 [33-55] | |
| De Bie et al. ^1^  (2020)  Netherlands  [RCT] | - Severe insufficiency 7 (6.3%) | Heart diseases | Acute hypoxemia | - ICU LOS, median [IQR] = Exp: 0.3 [0.3-0.6]; Con: 0.4 [0.3 - 0.7]. - ICU Mortality, n (%) = Exp: 3 (2.9); Con: 0. | - SAPS-2: 31 (29-39);  - APACHE IV: 41 (33-49). | - SAPS-2: 33 (28-39);  - APACHE IV: 38 (32-48). |
| Denault et al.^24^ (2020)  Canada  [RCT crossover] | - Hypertension, dyslipidemia, diabetes mellitus, HF, chronic kidney disease | NR | Acute hypoxemia | - LOS = NR - Mortality = NR | NR | |
| Eremenko et al.^2^  (2021)  Russia  [RCT] | NR | - Uncomplicated cardiac surgery - Exp, *n* = CABG: 18, valve surgery: 12, CABG + valve surgery: 2, aortic surgery: 8 - Con, *n* = CABG: 16, valve surgery: 12, CABG + valve surgery2, aortic surgery: 10 | Acute hypoxemia | - ICU LOS, median [IQR] = Exp: 1 [1-1]; Con: 1 [1-1]. - Hospitalar LOS, median [IQR] = Exp: 7 [6-11]; Con: 8 [7-12]. - Mortality = None patient died during the study period. | NR | NR |
| Hansen et al.^9^ (2018)  Denmark  [RCT crossover] | - *n* (%) = pneumonia: 6 (30) | - COPD | Chronic exacerbated hypoxemia | - Hospitalar LOS, mean (SD) = Exp: 8.1 (6) Con: 4.8 (2.6). - Mortality = NR. | GOLD, *n* (%)   - Group 1, 0 (0); - Group 2, 4 (20); - Group 3, 6 (30); - Group 4, 10 (50). | |
| Harper et al.^25^ (2021)  New Zealand  [RCT] | - Exp, *n* = asthma: 1, HF: 1, interstitial lung disease: 1, pneumonia: 4. - Con, *n* = asthma: 2, HF: 1, interstitial lung disease: 1; pulmonary embolism: 1, pneumonia: 2. | - Exp, *n* = asthma exacerbation: 2, COPD exacerbation: 3, HF: 1, interstitial lung disease: 1, pulmonary embolism: 1, pneumonia: 2. - Con, *n* = asthma exacerbation: 1, COPD exacerbation: 3, HF: 1, interstitial lung disease: 1, pneumonia: 4. | Acute hypoxemia | - Hospitalar LOS, mean (SD) = Exp: 8.1 (6) Con: 4.8 (2.6). - Mortality = NR. | NR | NR |
| Huynh Ky et al.^5^  (2017)  Canada  [RCT] | NR | - Acute phase of acute coronary syndrome. | NR | - LOS = NR. - Mortality = none patient died in all groups. | NR | NR |
| Johannigman et al.^13^  (2009)  USA  [RCT crossover] | NR | - *n* (%) = blunt trauma: 14 (93.3), penetrating trauma: 1 (6.7) | Acute hypoxemia | - LOS = NR. - Mortality = none patient died during the study period. | - Injury severity score, mean (SD) = 23.4 (3.9) | |
| Kobayashi et al.^6^  (2017)  Japan  [RCT] | NR | NR | NR | - LOS = NR - Mortality = NR | NR | NR |
| Komnov et al.^7^ (2023)  Russian  [RCT] | NR | Cardiac surgery (bypass, valve and ascending aortic surgery) | NR | - ICU LOS, median [IQR] = Exp: 1 [1-1]; Con: 1 [1-1]. - Mortality = none patient died in all groups. | - EuroSCORE II mean: 2.1 [CI 95%, 1.4-3.2] | - EuroSCORE II mean: 1.9 [CI 95%, 1.1-2.9] |
| L'Her et al.^3^ (2017)  France  [RCT] | - Exp, n (%) = COPD: 29 (31.5); immunodeficiency: 25 (26.9); do-not-intubated order: 7 (7.6); long-term oxygen therapy: 9 (9.8); home mechanical ventilation: 4 (4.3). - Con, *n* (%) = COPD: 36 (38.3); immunodeficiency: 22 (23.4); do-not-intubated order: 4 (4.3); long-term oxygen therapy: 8 (8.5); home MV: 1 (1.1). | - Hypoxaemic and hypercapnic respiratory failure | Acute hypoxemia | - ICU LOS, mean (SD) = Exp: 4.2 (3.2); Con: 4.7 (3.5).   Hospitalar LOS, mean (SD) = Exp: 9.2 (6.9); Con: 11.1 (7).   - 28-day mortality, *n* (%) = Exp: 10 (5.3); Con: 4 (2.4). | NR | NR |
| L'Her et al.^4^ (2021)  France  [RCT] | - Exp, *n* (%) = COPD: 7 (7.3), alcohol abuse: 15 (17.9), diabetes: 13 (15.5), arterial hypertension: 35 (41.2), coronaropathy: 8 (9.5), cardiac insufficiency: 2 (2.4), respiratory allergy: 3 (3.6), smokers: 53 (63.1), asthma: 2 (2.4), renal insufficiency: 6 (7.1). - Con, *n* (%) = COPD: 10 (9.7), alcohol abuse: 11 (12.1), diabetes: 13 (14.1), arterial hypertension: 32 (34.8), coronaropathy: 9 (9.8), respiratory allergy: 15 (16.5), smokers: 48 (52.7), asthma: 5 (5.5), long term oxygen therapy: 1 (1.1), renal insufficiency: 8 (8.7). | - Exp, *n* = thoracic surgery: 28, abdominal surgery: 67. - Con, *n* = thoracic surgery: 29, abdominal surgery: 74. | Acute hypoxemia | - Hospitalar LOS (day 28), mean (SD) = Exp: 12.5 (12.4); Con: 13.3 (11.3). - 28-day mortality, *n* (%) = Exp: 3 (3.2); Con: 1 (1.0). | - ARISCAT risk class  - Moderate risk (26-44), *points* (%) = 72 (75)  - ARISCAT risk class  - High risk (≥ 45), *points* (%) = 24 (25) | - ARISCAT risk class  - Moderate risk (26-44), *points* (%) = 78 (75.7)  - ARISCAT risk class  - High risk (≥ 45), *points* (%) = 25 (24.3) |
| Lellouche et al.^17^  (2013)  Canada  [RCT] | - Exp, *n* (%) = diabetes mellitus: 5 (16.7), arterial hypertension: 20 (66.7), atrial fibrillation: 1 (3.3), recent myocardial infarction: 3 (10), NYHA III/IV: 8 (26.7), previous renal failure: 1 (3.3). - Con, *n* (%) = diabetes mellitus: 10 (33.3), arterial hypertension: 23 (76.7), atrial fibrillation: 3 (10), myocardial infarction: 7 (23.3), NYHA III/IV: 12 (40), COPD: 4 (13.3). | Cardiac surgery | Acute hypoxemia | - LOS = NR - Mortality = NR | - Parsonnet score,  mean (SD) = 10.2 (7.8) | - Parsonnet score, mean (SD) = 9.0 (8.5) |
| Lellouche et al.^26^  (2016)  Canada  [RCT] | NR | Acute exacerbation of COPD | Chronic exacerbated hypoxemia | - Hospitalar LOS, mean (SD) = Exp: 6.4 (4.3); Con: 9.5 ( 6). - Mortality = NR | Spirometry during hospital stay:  - FEV_1_, L (SD) = 1.0 (0.5)  - FEV_1_, %_predicted_ (SD) = 36 (19)  - FEV_1_/FVC, % (SD) = 45 (12) | Spirometry during hospital stay:   - FEV_1_, L (SD) = 1.0 (0.5) - FEV_1_, %_predicted_ (SD) = 38 (24) - FEV_1_/FVC, % (SD) = 49 (13) |
| Roca et al.^14^  (2022)  Spain  [RCT crossover] | - *n* (%) = hypertension: 11 (24), diabetes: 7 (15.5), immunosuppression: 10 (22.2), chronic respiratory disease: 6 (13.3). | - *n* (%) = COVID-19 pneumonia: 36 (80), non-COVID pneumonia: 5 (11.1), other: 4 (8.8) | Acute hypoxemia | - ICU LOS, mean (SD) = Exp: 9.14 (5.95); Con: 11.35 (6.58). - Hospitalar LOS, mean (SD) = Exp: 16.36 (10.93); Con: 27.7 (21.77). - ICU Mortality, *n* (%) = 2 (4.4). - Hospital mortality, *n* (%) = 4 (4.4). | - SAPS III, median [IQR] = 45 [39-52]; - APACHE II, median [IQR] = 8.5 [5.0-15.3]; - SOFA, median [IQR] = 3 [3-3]. | |

*Abbreviations:* ADCPD = acute decompensation in chronic pulmonary disease; APACHE II = acute physiology and chronic health evaluation; ARDS = acute respiratory distress syndrome; ARF = acute respiratory failure; ARISCAT = assess respiratory risk in surgical patients in catalonia; bpm = beats per minute; CABG = coronary artery bypass graft; CI = confidence interval; Con = control; COPD = chronic obstructive pulmonary disease; COVID-19 = coronavirus disease 19; Exp = experimental; FEV_1_ = forced expiratory volume in one second; FVC = forced vital capacity; GOLD = global initiative for obstructive lung disease; HF = heart failure; ICU = intensive care unit; IQR = interquartile range; L = Liter; LOS = length of stay; LVEF = left ventricular ejection fraction; MV = mechanical ventilation; NR = not reported; NYHA = New York heart association; PEtCO_2_ = end-tidal carbon dioxide tension; RCT = randomized controlled trial; SAPS III = simplified acute physiology score III; SD = standard deviation; SOFA = sequential organ failure assessment; USA = United States of America.

**S10 Table.** Funding and sponsorship

| **First author (year)** | **Funding** | **Sponsorship** | **Authors' Conflicts of interest** |
| --- | --- | --- | --- |
| Arnal et al.^10^  (2010) | Hamilton Medical | NR | NR |
| Arnal et al.^16^  (2012) | NR | Hamilton Medical | NR |
| Arnal et al.^22^  (2018) | This study was initiated by the center Hospitalier intercommunal de toulon la seyne sur Mer and supported by an unrestricted grant from Hamilton Medical | Hamilton Medical | “J.M.A is a part time employee of Hamilton Medical, working as a medical research manager; A.G. was supported by Hamilton Medical to present the results of this study at the ESICM meeting in Barcelona in 2014. A.G. received some honoraria for educational activities from Hamilton Medical; D.N. is a full-time employee of Hamilton Medical, working as the manager of applied research and new technology; T.L. is a full time employee of Hamilton Medical, working as a medical research engineer.” |
| Bialais et al.^23^  (2016) | NR | Hamilton Medical (providing material support) | “M.W. was employed full-time at Hamilton Medical AG when the study was designed. J.M. is a research engineer and D.N. is manager of research at Hamilton Medical AG. All other authors certify that they have no conflict of interest regarding the material discussed in this manuscript.” |
| Chelly et al.^11^  (2020) | The study was integrally supported by the Groupe Hospitalier Sud Ile de France (Melun, France) | NR | “J.C. and S.J., received fees from Hamilton Medical for lecturing.” |
| Clavieras et al.^12^  (2013) | NR | NR | “M.W. was the director of the Medical Research Department of Hamilton Medical when the study was designed. M.W. is co-sharing a patent on Intellivent. (WO/2007/085110 and WO/2007/085108).” |
| De Bie et al.^1^  (2020) | Catharina Hospital Eindhoven; Amsterdam University Medical Centers; Hospital Israelita Albert Einstein. | Catharina Ziekenhuis Eindhoven | “A.J.R.D.B’s PhD research is funded by the Impuls-2 project, a collaboration of Catharina Hospital Eindhoven, Eindhoven University of Technology, and Philips Research. In 2018, A.J.R.D.B., and M.J.S. attended a workshop organized by Hamilton, in which expenses for lodging were covered for all invited experts, participants from abroad had their travel expenses reimbursed, and speakers received a speaker’s fee of CHF 800-. A.R.B. received travel support from C.S.L. Behring to visit Network for the Advancement of Patient Blood Management, Haemostasis and Thrombosis (NATA) 2015. ARB is clinical consultant for Philips Research in Eindhoven, Netherlands, since January 2016. E.H.M.K., is clinical consultant for Philips Research since January 2016.” |
| Denault et al.^24^  (2020) | Support was provided solely from institutional sources. | NR | “M.S. is a shareholder and administrator of Oxynov, the company that developed and is commercializing FreeO2. François Lellouche is a co-founder, shareholder, and administrator of Oxynov. He co-invented the FreeO2 device used in this study. F.L. receives financial support from the Canada Foundation for Innovation (Research program for automation of ventilation and oxygenation). M.H.D., C.R., P.A.B. and S.S. declare no competing interests.” |
| Eremenko et al.^2^  (2021) | NR | NR | “The authors' declare no conflicts of interest”. |
| Hansen et al.^9^  (2018) | NR | NR | “E.F.H. and J.D.H. are co-inventors of O2matic^®^ and both have participated in the development and testing of the device since 2011. The partnership, which was built during the development funded by Innovation Fund Denmark, formed a new company (O2matic Ltd., Herlev, Denmark) and both E.F.H. and J.D.H. participate as shareholders in O2matic Ltd. J.V. is supported by the NIHR Manchester  Biomedical Research Center.” |
| Harper et al.^25^  (2021) | This study was funded by Fisher and Paykel Healthcare, Auckland, New Zealand. | NR | “J.H., reports personal fees from Fisher and Paykel Healthcare, outside the submitted work. R.B., reports a grant from Fisher and Paykel Healthcare to undertake this study.” |
| Huynh et al.^5^  (2017) | NR | NR | “Co-founder of the Oxynov company that develops the FreeO2 system.” |
| L'Her et al.^3^  (2017) | Programme Hospitalier de Recherche Clinique National 2010 from the French Ministry of Health for the French centers and by the Ministry of Finance in Québec for Canadian centers. | “The study was sponsored by Brest University Hospital, which was responsible for data management and control.” | “E.L.H. reports grant from Canadian Foundation for innovation, other from FRSQ (Fond de Recherche en Santé du Québec), other from Oxynov, during the conduct of the study; other from Oxynov, outside the submitted work; In addition, F.L. has a patent Method and device for delivering oxygen pending. E.L.H. reports other from Oxynov, during the conduct of the study; other from Oxynov, outside the submitted work; In addition, E.L.H. has a patent Method and device for delivering oxygen pending.” |
| L'Her et al.^4^  (2021) | This trial was funded by the French Ministry of Health obtained in 2014 from a regional hospital clinical research programme (Programme Hospitalier de Recherche Clinique Interrégional HUGO 2012-199). The sponsors had no role in the study design and conduct; the collection, management, analysis and interpretation of the data; or the preparation and approval of the manuscript. Funding information for this article has been deposited with the Crossref Funder Registry. | NR | “E.L.H. reports other from Oxynov, during the conduct of the study; personal fees from Smiths Medical, personal fees and other from GE Healthcare, grants and personal fees from Sedana Medical, outside the submitted work. In addition, E.L.H. has a patent method and device for delivering oxygen licensed to Oxynov. S.J. reports personal fees from Drager, Fisher-Paykel, Baxter, Fresenius-Xenios and Medtronic, during the conduct of the study. E.F. reports consulting fees from Drager Medical, GE Healthcare, Orion Pharma and Edwards Lifesciences, lecture fees from Fresenius Kabi and Getinge, and non-financial support from Fisher and Paykel Healthcare, during the conduct of the study. V.P. reports others from OxyNov, during the conduct of the study. D.V., C.J., T.K., P.B., M.C., and B.H. have nothing to disclose.” |
| Lellouche et al.^17^  (2013) | The Fond de Recherche en Santé´ du Québec and Hamilton Medical provided a restricted grant of CAD 60,000 to conduct the study and provided ventilators for the study. | Hamilton Medical | “M.W. was head of medical research at Hamilton Medical Company when the study was performed; he is now Medical Director of GE Healthcare.” |
| Lellouche et al.^26^  (2016) | The Fond de Recherche en Santé du Québec contributes to F.L's salary for research activities (clinical research scholar) and to the research assistant’s salary (clinical research grant). | NR | “F.L. and E.L.H., are the co-inventors of the FreeO2 system and made the first prototypes with the engineering department of Laval University. F.L. and E.L.H., are the cofounders of a Laval University spin-off research and development company (OxyNov) to develop automated systems for respiratory support. F.M., holds a GlaxoSmithKline/Canadian Institutes of Health Research chair on COPD at Laval University. F.M. and Y.L., participate in Innovair, a company that owns shares in OxyNov, the owner of the FreeO2 device. P.A.B. and M.R., have no financial interests that may be relevant to the submitted work. The authors report no other conflicts of interest in this work.” |
| Roca et al.^14^  (2022) | This study is partially supported by a research grant from Hamilton Medical AG. For each patient, an anonymized file containing the recorded data from the high-flow device was sent to Hamilton Medical AG, where these data were transformed into a database of raw data and sent back to the investigators for further analysis. Hamilton Medical AG did not have a role in statistical analysis beyond this point or data interpretation. | NR | “O.R., discloses a research grant from Hamilton Medical AG and speaker fees from Hamilton Medical AG, Ambu, Aerogen Ltd, and Fisher & Paykel Healthcare Ltd, and non-financial research support from Timpel and Massimo Corporation. R.F., discloses personal fees from MSD, Pfizer, Shionogi, Gilead, Grifols, Menarini, and GSK. M.J.S., discloses speaker fees from Hamilton Medical AG. JDR discloses travel expenses from Fisher & Paykel Healthcare Ltd. All other authors disclose no conflict of interest.” |

*Abbreviations:* NR = not reported.

**S11 Table.** Summary of the findings

| **Certainty assessment** | | | | | | | **Summary of findings** | | | | |
| --- | --- | --- | --- | --- | --- | --- | --- | --- | --- | --- | --- |
| **Participants**  **(studies)**  **Follow-up** | **Risk of bias** | **Inconsistency** | **Indirectness** | **Imprecision** | **Publication bias** | **Overall certainty of evidence** | **Study events rates (%)** | | **Relative effect**  **(95% CI)** | **Anticipated absolute effects** | |
|  |  |  |  |  |  |  | **With manual oxygen titration** | **With closed-loop oxygen titration** |  | **Risk with manual oxygen titration** | **Risk difference with closed-loop oxygen titration** |

**Time spent in SpO_2_ target**

| *n* = 771  (12 RCTs) | Not serious | Serious^a^ | Not serious | Not serious | None | ⨁⨁⨁◯  **Moderate** | Seven studies^1,3,4,14,23,25,26^ were included in the quantitative analysis and presented a difference with a closed-loop of MD 25.47% higher (19.93 higher to 31 higher). The risk with manual oxygen titration ranged from 19.7 to 30% of time on average. Four crossover studies^9,11,13,15^ were not included due to the absence of reported data of the crossover first period. One study^17^ was not included due to the absence of information of all patients included to change mean (SD) data from minutes to percentage of time. The authors evaluated the time, in minutes, spent in the optimal zone with a mean of 192 (SD 52) in the closed-loop group and 25 (SD 124) in the control group. |
| --- | --- | --- | --- | --- | --- | --- | --- |

**Time for oxygen weaning**

| *n* = 436  (3 RCTs) | Not serious | Not serious | Not serious | Serious ^b^ | None | ⨁⨁⨁◯  **Moderate** | In one study^3^ the intervention was applied for a 3-hour period and the mean time spent in oxygen support was 5.6 (SD 5.4) days in the closed-loop group and 7.1 (SD 6.3) days in the control group. One study^4^ evaluated in 181 participants the time spent in the O_2_ support in minutes until 3-days, and the closed-loop group weaned from oxygen slightly faster than the control group, with mean of 3,146 (SD 1,225) and mean of 3,506 (SD 982), respectively. In one study^26^ the intervention lasted 7-days and the mean time spent in oxygen support was 4.0 (SD 2.1) days in the closed-loop group and 5.8 (SD 9.9) days in the control group. |
| --- | --- | --- | --- | --- | --- | --- | --- |

**Length of stay**

| *n* = 973  (10 RCTs) | Not serious | Not serious | Not serious | Serious ^c^ | None | ⨁⨁⨁◯  **Moderate** | Eight^2-4,14,18,22,25,26^ studies reported data of hospital length of stay and seven^1-3,7,14,18,22^ of ICU length of stay. Intervention duration varied from 2-hours to total hospital length of stay. One study^14^ presented a crossover design of clinical trials. |
| --- | --- | --- | --- | --- | --- | --- | --- |

**Cost**

| *n* = 47  (1 RCT) | Not serious | Not serious | Not serious | Very  serious ^d^ | None | ⨁⨁◯◯  **Low** | One study^8^ (in one study^26^), 47 participants reported hospitalization cost structure per patient at 180 days in 2015 Canadian dollars (CAD) 6,098.30 in the closed-loop group and CAD 8,140.10 in the control group. The mean difference of CAD 2,959.71 within groups, representing a 20.7% of cost reduction in the intervention group. |
| --- | --- | --- | --- | --- | --- | --- | --- |

**Adverse events**

| *n* = 1,115  (14 RCTs) | Serious^e^ | Serious^f^ | Not serious | Serious^g^ | None | ⨁◯◯◯  **Very low** | Fourteen studies^2-4,6,9-13,16,17,23,26^ reported quantitative or qualitative analyses of adverse events. Of those, six^9-13,16^ were crossover trials. A wide variety of adverse events were reported in the studies, without a standardization of the type and gravity of the events (e.g., hypoxemia; barotrauma or other events related to mechanical ventilation; bradycardia; premature interruption of intervention; asynchrony; and technical issues) |
| --- | --- | --- | --- | --- | --- | --- | --- |

**Mortality**

| *n* = 859  (10 RCTs) | Serious^e^ | Not serious | Not serious | Serious^c^ | None | ⨁⨁◯◯  **Low** | Hospital mortality was reported by seven studies^2,5,7,12-14,23^, of which three studies^12-14^ presented a crossover design. ICU mortality was investigated in five studies^1,4,14,22,23^ (four RCTs^1,4,22,23^ and one crossover study^14^), of which, three studies^4,22,23^ reported ICU mortality until 30-day of length of stay. Intervention duration varied from 3-hours to total hospital length of stay. |
| --- | --- | --- | --- | --- | --- | --- | --- |

**Health professional workload**

| *n* = 327  (7 RCTs) | Not serious | Serious^h^ | Not serious | Serious^i^ | None | ⨁⨁◯◯  **Low** | Four studies^7,22,23,25^ produced as a result of risk with closed-loop is MD 4.98 lower (7.28 lower to 2.61 lower). The risk with manual oxygen titration for healthcare workload ranged from 4.8 to 13 adjusts on average. Two crossover studies^14,16^ investigated this outcome; however, they were not included in the meta-analysis. One study^16^ reported no adjustments in both groups and the first period data was not available. One study^14^ reported the results as manual adjustments per hour with a mean of 0 (SD 0) in the closed-loop group and 0.50 (SD 0.54) in the control group, considering the data of the first period. One study^2^ investigated this outcome as adjustments per patient and sent the results in median [IQR] = 0 [0 to 0] in the closed-loop group *versus* 4 [3 to 5] in the control group. |
| --- | --- | --- | --- | --- | --- | --- | --- |

*Abbreviations:* CI = confidence interval; ICU = intensive care unit; IQR = interquartile range; MD = mean difference; RCT = randomized controlled trial; RR = risk ratio; SpO_2_ = peripheral capillary oxygen saturation.

#### **Explanations:**

#### a. Downgraded one level due to serious unexplained inconsistency (large heterogeneity in the subgroup analysis *I^2^* = 68%, *p* value [0.04])

#### b. Downgraded one level due to serious imprecision (few trials)

#### c. Downgraded one level due to serious imprecision (large confidence interval of the individual trials)

#### d. Downgraded two levels due to very serious imprecision (few events)

#### e. Downgraded one level due to methodological limitations.

#### f. Downgraded one level due to serious unexplained inconsistency (large heterogeneity in the analysis *I^2^* = 87%, *p* value = 0.005)

#### g. Downgraded one level due to serious imprecision (large confidence interval and few events)

#### h. Downgraded one level due to serious inconsistency (large heterogeneity in the subgroup analysis *I^2^* = 75%, *p* value = 0.008)

#### i. Downgraded one level due to serious imprecision (few participants)

####

**S12 Table.** Quantitative and qualitative data of adverse events of the included trials

| **First author**  **(year)** | **Adverse Events Definition** | **Unit of measure** | **Adverse events** |
| --- | --- | --- | --- |
| Arnal et al.^10^  (2010) | - Major safety issues (no details of the type of event) | NR | - No patient was removed from the intervention group for major safety issues. |
| Arnal et al.^16^  (2012) | - Premature interruption of INTELLiVENT^®^-ASV due to SpO_2_ decreased below 85 % for more than 2 min, or if Vt delivered was over 12 mL/kg PBW for more than 2 min; or to switch to a conventional mode for any clinical reason judged by the clinician as potentially harmful for the patient. | Number of events | - There was no safety issue requiring premature interruption of INTELLiVENT^®^-ASV. |
| Bialais et al.^23^  (2016) | - Barotrauma or other adverse event related to mechanical ventilation. | Number of events | - No case of barotrauma or other adverse event related to mechanical ventilation was observed. |
| Chelly et al.^11^  (2020) | - Occurrence of major adverse events (accidental endotracheal tube removal, bradycardia lower than 40 bpm, or cardiac arrest) during the DNP. With the exception of one episode of bradycardia (< 40 bpm) in one patient during the automated ventilation period, no major adverse events occurred during DNPs in either period. | Number of events | - Exp = bradycardia (< 40 bpm), accidental disconnection of the ventilator occurred in 18 (7%). - Con, *n* (%) = 16 (6) patients during the automated ventilation and conventional ventilation periods. |
| Clavieras et al.^12^  (2013) | - Safety issues requiring premature interruption of intervention for the studied patients. | Number of events | - Exp, *n* = none (0) - Con, *n* = none (0) |
| Eremenko et al.^2^  (2021) | - Undesirable events: asynchrony between patient and ventilator; shortly anxiety (tachycardia, tachypnea, knocking on the bed during awakening and weaning process) | Number of patients | - Cases of asynchrony between patient and ventilator  - Exp, *n* (%) = 4 (10); Con, *n* (%) = 8 (20)  - Shortly anxiety  - Exp, *n* (%) = 5 (12.5); Con, *n* (%) = 9 (22.5) |
| Hansen et al.^9^  (2018) | - Trouble with device | Number of events | - In one instance audible and visible alarms for no power supply and low battery were ignored resulting in shutdown of oxygen supply after 2 hours of battery mode, which is in accordance with specifications for battery durability in O2matic. The patient was on low-dose oxygen and no harm was reported, but equipment alarms were afterward adjusted to flash on the screen if there is a lack of power supply and warn that there could be imminent shutdown. No other safety issues were observed. |
| Harper et al.^25^  (2021) | - Adverse events | Number of events | - There was one MET call, one ICU admission and one death involving the same participant in the automatic titration group. This was considered to be a serious adverse event which was reviewed by the DSMC, and was determined to be unrelated to the study intervention. There was a device fault for a different participant in the automatic titration group which resulted in an unexpected shutdown of closed-loop control after 19.7 hours, and was also reviewed by the DSMC. This did not lead to any adverse effects for the participant and the fault was remedied by a software update prior to further participant recruitment. |
| Johannigman et al.^13^  (2009) | - Episodes with SpO_2_ < 88% | Number of events | - Exp, *n* = 15 - Con, *n* = 16 |
| Kobayashi et al.^6^  (2017) | - Safety issues (not detailed) | Number of events | - No safety issues occurred. |
| L'Her et al.^3^  (2017) | - Serious adverse events and other adverse events (no details of the type of event) | Number of participants | - Serious adverse events   - Exp, *n* (%) = 18 (9.6); Con, *n* (%) = 15 (8)   - Other adverse events:   - Exp, *n* (%) = 43 (23); Con, *n* (%) = 32 (17.1) |
| L'Her et al.^4^  (2021) | - Hypoxaemia for >10% of the time during the 3 days of recording | Number of participants | - Exp, *n* = 0 - Con, *n* = 25 |
| Lellouche et al.^17^  (2013) | - 'not acceptable' zone of ventilation | Number of participants | - Exp, *n* (%) = 4 (13) - Con, *n* (%) = 13 (43) |
| Lellouche et al.^26^ (2016) | - Safety / Oxygen delivery interruption with FreeO_2_ | Number of events | - Few technical issues occurred with end-tidal CO_2_ monitoring, but none concerned the oxygen delivery valve, and this was not associated with safety issues. |

*Abbreviations:* ASV = adaptive support ventilation; bpm = beats per minute; Con = control; CO_2_ = carbon dioxide; DNP = daily nursing procedures; DSMC = data safety monitoring committee; Exp = experimental; ICU = intensive care unit; MET = medical emergency team; NR = not reported; PBW = predicted body weight; SpO_2_ = peripheral oxygen saturation.

**e-References**

1. [De Bie AJR, Neto AS, van Meenen DM, et al. Fully automated postoperative ventilation in cardiac surgery patients: a randomised clinical trial. *Br J Anaesth*. 2020;125(5):739-749. doi:](http://paperpile.com/b/PJihzf/nbU7Z)[10.1016/j.bja.2020.06.037](http://dx.doi.org/10.1016/j.bja.2020.06.037)

2. Eremenko А.А., Komnov R.D., Titov P.А., Gerasimenko S.А., Chakal D.А. Comparing the Intellivent-ASV® Mode with Conventional Ventilation Modes during Weaning after Uncomplicated Cardiac Surgery. Messenger of Anesthesiology and Resuscitation. 2021;18(3):36-45. (In Russ.) <https://doi.org/10.21292/2078-5658-2021-18-3-36-45>

3. [L’Her E, Dias P, Gouillou M, et al. Automatic versus manual oxygen administration in the emergency department. *Eur Respir J*. 2017;50(1). doi:](http://paperpile.com/b/PJihzf/4Ji4O)[10.1183/13993003.02552-2016](http://dx.doi.org/10.1183/13993003.02552-2016)

4. [L’Her E, Jaber S, Verzilli D, et al. Automated closed-loop versus standard manual oxygen administration after major abdominal or thoracic surgery: an international multicentre randomised controlled study. *Eur Respir J*. 2021;57(1). doi:](http://paperpile.com/b/PJihzf/3x81i)[10.1183/13993003.00182-2020](http://dx.doi.org/10.1183/13993003.00182-2020)

5. Huynh Ky MT, Bouchard P-A, Morin J, L'Her E, Sarrazin J-F, Lellouche F. Closed-Loop Adjustment of Oxygen Flowrate with FreeO2 in Patients with Acute Coronary Syndrome: Comparison of Automated Titration with FreeO2 (Set at Two SpO2 Target) and of Manual Titration. A Randomized Controlled Study. Am J Respir Crit Care Med. 2017;195:A3766.

6. Kobayashi T, Onodera Y, Suzuki H, Nakane M, Kawamae K. Manual ASV vs INTELLiVENT-ASV for the patients after cardiac surgery - are automated ventilators better for the patients? Intensive Care Medicine Experimental. ESICM LIVES 2017. 2017;5(Suppl 2):0749. <https://doi.org/10.1186/s40635-017-0151-4>.

7. Komnov R, Eremenko A, Alferova A, Riabova D, Titov P, Urbanov A, Fominukh M, Gerasimenko S. Benefits of fully closed loop ventilation modes in patients with body mass index > 35 kg/m2. Critical Care. 42nd International Symposium on Intensive Care & Emergency Medicine. 2023;27(S1):P023. <https://doi.org/10.1186/s13054-023-04377-x>

8. [Poder TG, Kouakou CRC, Bouchard PA, et al. Cost-effectiveness of FreeO in patients with chronic obstructive pulmonary disease hospitalised for acute exacerbations: analysis of a pilot study in Quebec. *BMJ Open*. 2018;8(1):e018835. doi:](http://paperpile.com/b/PJihzf/SdUJV)[10.1136/bmjopen-2017-018835](http://dx.doi.org/10.1136/bmjopen-2017-018835)

9. [Hansen EF, Hove JD, Bech CS, Jensen JUS, Kallemose T, Vestbo J. Automated oxygen control with O2matic during admission with exacerbation of COPD. *Int J Chron Obstruct Pulmon Dis*. 2018;13:3997-4003. doi:](http://paperpile.com/b/PJihzf/wsL06)[10.2147/COPD.S183762](http://dx.doi.org/10.2147/COPD.S183762)

10. [Arnal JM, Wysocki M, Demory D, et al. Prospective Randomized Cross-over Controlled Study Comparing Adaptive Support Ventilation (ASV) And A Fully Close Loop Control Solution (Intellivent®) In Adult ICU Patients With Acute Respiratory Failure. *B42 INVASIVE MECHANICAL VENTILATION: SOUP TO NUTS*. Published online 2010. doi:](http://paperpile.com/b/PJihzf/T3CXL)[10.1164/ajrccm-conference.2010.181.1_meetingabstracts.a3004](http://dx.doi.org/10.1164/ajrccm-conference.2010.181.1_meetingabstracts.a3004)

11. [Chelly J, Mazerand S, Jochmans S, et al. Automated vs. conventional ventilation in the ICU: a randomized controlled crossover trial comparing blood oxygen saturation during daily nursing procedures (I-NURSING). *Crit Care*. 2020;24(1):453. doi:](http://paperpile.com/b/PJihzf/WPOWO)[10.1186/s13054-020-03155-3](http://dx.doi.org/10.1186/s13054-020-03155-3)

12. [Clavieras N, Wysocki M, Coisel Y, et al. Prospective Randomized Crossover Study of a New Closed-loop Control System versus Pressure Support during Weaning from Mechanical Ventilation. *Anesthesiology*. 2013;119(3):631-641. doi:](http://paperpile.com/b/PJihzf/a6TNR)[10.1097/ALN.0b013e3182952608](http://dx.doi.org/10.1097/ALN.0b013e3182952608)

13. [Johannigman JA, Branson R, Lecroy D, Beck G. Autonomous Control of Inspired Oxygen Concentration During Mechanical Ventilation of the Critically Injured Trauma Patient. *J Trauma Acute Care Surg*. 2009;66(2):386. doi:](http://paperpile.com/b/PJihzf/USZdW)[10.1097/TA.0b013e318197a4bb](http://dx.doi.org/10.1097/TA.0b013e318197a4bb)

14. [Roca O, Caritg O, Santafé M, et al. Closed-loop oxygen control improves oxygen therapy in acute hypoxemic respiratory failure patients under high flow nasal oxygen: a randomized cross-over study (the HILOOP study). *Crit Care*. 2022;26(1):108. doi:](http://paperpile.com/b/PJihzf/JuGou)[10.1186/s13054-022-03970-w](http://dx.doi.org/10.1186/s13054-022-03970-w)

15. Buiteman-Kruizinga LA, Tsonas AM, Botta M, Guseva AA, Serpa NA, Schultz M, Paulus F. The Effect of INTELLiVENT–ASV on Oxygenation, FiO2 and PEEP in Critically Ill Invasively Ventilated Patients. Intensive Care Medicine Experimental. ESICM LIVES 2022: part 1. 2022;10(2):000489. <https://doi.org/10.1186/s40635-022-00468-1>

16. [Arnal JM, Wysocki M, Novotni D, et al. Safety and efficacy of a fully closed-loop control ventilation (IntelliVent-ASV®) in sedated ICU patients with acute respiratory failure: a prospective randomized crossover study. *Intensive Care Med*. 2012;38(5):781-787. doi:](http://paperpile.com/b/PJihzf/6702g)[10.1007/s00134-012-2548-6](http://dx.doi.org/10.1007/s00134-012-2548-6)

17. [Lellouche F, Bouchard PA, Simard S, L’Her E, Wysocki M. Evaluation of fully automated ventilation: a randomized controlled study in post-cardiac surgery patients. *Intensive Care Med*. 2013;39(3):463-471. doi:](http://paperpile.com/b/PJihzf/nXeDQ)[10.1007/s00134-012-2799-2](http://dx.doi.org/10.1007/s00134-012-2799-2)

18. Bialais E, Vignaux L, Wittebole X, Novotni D, Meyer J, Wysocki M, Sottiaux T, Reychler G, Roeseler J, Laterre P, Hantson P. Comparison of an entirely automated ventilation mode, Intellivent-ASV, with conventional ventilation in ARDS patients: a 48-hour study. Critical Care. 2013;17(Suppl 2):P98. DOI: 10.1186/cc120236.

19. Bialais E, Vignaux L, Wittebole X, Novotni D, Meyer J, et. al. Safety and Efficacy of ventilation delivered by a full automated mode (Intellivent-ASV): a randomized controlled study. 26th Annual Congress ESICM ((France) Paris, du 05/10/2013 au 09/10/2013). In: Intensive Care Medicine, Vol. 39, no. Suppl 2, p. S248 (2013)

20. Malika M. Continuous control of arterial oxygenation in mechanically ventilated patients. Ann Intensive Care. 2018;8(Suppl 1):F-83. DOI: 10.1186/s13613-017-0345-7.

21. Ye Z, Xu J, Zhang C, Shi KJ, Sun F, Mu XW. The efficacy of IntelliVent-ASV mode in post-cardiac surgery patients. J Am Coll Cardiol. 2016;68(16 Suppl S).

22. [Arnal JM, Garnero A, Novotni D, et al. Closed loop ventilation mode in Intensive Care Unit: a randomized controlled clinical trial comparing the numbers of manual ventilator setting changes. *Minerva Anestesiol*. 2018;84(1):58-67. doi:](http://paperpile.com/b/PJihzf/HTYWm)[10.23736/S0375-9393.17.11963-2](http://dx.doi.org/10.23736/S0375-9393.17.11963-2)

23. [Bialais E, Wittebole X, Vignaux L, et al. Closed-loop ventilation mode (IntelliVent®-ASV) in intensive care unit: a randomized trial. *Minerva Anestesiol*. 2016;82(6):657-668.](http://paperpile.com/b/PJihzf/6p9uL) <https://www.ncbi.nlm.nih.gov/pubmed/26957117>

24. Denault MH, Ruel C, Simon M, Bouchard PA, Simard S, Lellouche F. Evaluation of hyperoxia-induced hypercapnia in obese patients after cardiac surgery: a randomized crossover comparison of conservative and liberal oxygen administration. Can J Anaesth. 2020 Feb;67(2):194-202. English. doi: 10.1007/s12630-019-01500-x. Epub 2019 Oct 24. PMID: 31650500.

25. [Harper J, Kearns N, Bird G, et al. Automatic versus manual oxygen titration using a novel nasal high-flow device in medical inpatients with an acute illness: a randomised controlled trial. *BMJ Open Respir Res*. 2021;8(1). doi:](http://paperpile.com/b/PJihzf/NSnQg)[10.1136/bmjresp-2020-000843](http://dx.doi.org/10.1136/bmjresp-2020-000843)

26. [Lellouche F, Bouchard PA, Roberge M, et al. Automated oxygen titration and weaning with FreeO_2_ in patients with acute exacerbation of COPD: a pilot randomized trial. *COPD*. 2016;11(1):1983-1990. doi:](http://paperpile.com/b/PJihzf/9cTc2)[10.2147/COPD.S112820](http://dx.doi.org/10.2147/COPD.S112820)
