# Supplementary material for: Closed-loop oxygen control for critically ill patients––A systematic review and meta-analysis
Source: PLoS One. 2024 Jun 12;19(6):e0304745. doi: 10.1371/journal.pone.0304745 (PMC11168613; doi:10.1371/journal.pone.0304745)
Supplement: Attachment — Submitted filename: 0. [PLoS One - Major Revision] Rebuttal letter.docx [file pone.0304745.s002.docx]

To:	Prof. Vijay Hadda

Editor

PLoS One

From:	Caroline Gomes Mól, PT PhD

Department of Critical Care Medicine

Hospital Israelita Albert Einstein

São Paulo, Brazil

**Revised version of [PONE-D-24-04130] Closed-loop oxygen control for critically ill patients––A systematic review and meta-analysis**

April 18, 2024

Dear prof. Vijay Hadda, dear editor,

We sincerely appreciate the opportunity to submit the revised version of our manuscript entitled ‘Closed-loop Oxygen Control for Critically Ill Patients: A Systematic Review and Meta-analysis’.

Your comments and those of the respected reviewers were very helpful for revising the work, we have meticulously implemented changes to enhance the quality and clarity of our manuscript.

Below, we provide a detailed point-by-point response to the reviewer’s comments.

We look forward to your response, and also the responses from the reviewers if they get a second chance to review our work.

Kindest regards, and on behalf of all coauthors,

Caroline Gomes Mól

**ACADEMIC EDITOR:**

1. The language need thorough check for grammar and syntax.

**Thank you for your comment. We have carefully reviewed both the manuscript and supporting information, conducting a thorough check for grammar and syntax.**

2. Also, it would be better if main results can be presented as main text, instead of supplementary.

**Thank you for your comment. As suggested, we have transferred certain topics and figures from the supplementary material to the main text, as outlined below:**

- **[Page 9; line 10] S1 Appendix. Overall risk of bias for each planned outcome.**
- **[Page 9; line 13; Figure 2; ‘5. [PLoS One - Major Revision] Fig 2 Rob RCT’] S1 Fig. Risk of bias of RCT included studies.**
- **[Page 9; line 24; Figure 3; ‘6. [PLoS One - Major Revision] Fig 3 Rob RCT crossover’] S2 Fig. Risk of bias of crossover included studies.**
- **[Page 11; line 3] S3 Appendix. Summary of studies not included in the quantitative analyses.**
- **[Page 12; line 15; ‘7. [PLoS One - Major Revision] Fig 5’] S10 Fig. Forest plot of healthcare professionals' workload.**

**Reviewer 1**

1. Most studies have a very short duration of intervention ranging for only few hours which will make it difficult to generalize the results for patients requiring oxygen for prolonged duration.

**Thank you for your comment. This is a very valid and important comment. We added this limitation to the limitations section of the Discussion of the revised version of the manuscript, as follows:**

- **[Page 16; line 13] *‘It is important to emphasize that most studies in this meta-analysis featured brief intervention periods, ranging from only a few hours to 24 hours. This limitation diminishes the generalizability of the results to patients necessitating prolonged oxygen therapy, typically those in the most critical condition.’***

2. Exclusion criterion in various studies can be included.

**We appreciate your suggestions. Exclusion criteria of studies are now provided in table 1 as follows:**

**Table 1.** Summary of included studies

| **Author**  **(year)**  **Country** | **Study type** | **Participants** | **Intervention** | |
| --- | --- | --- | --- | --- |
|  |  |  | **Exp** | **Con** |
| Arnal et al.^34^  (2010)  France | RCT crossover | - n = 43  - Age, mean (SD), *y* = 64 (15)  - Gender = NR  - Eligibility = NR  - Exclusion criteria = NR | - IMV with closed-loop control of oxygen (INTELLiVENT^®^–ASV)  - Duration = 2-hours  - Follow-up = NR | - IMV with manual control of oxygen (ASV mode)  - Duration = 2-hours  - Follow-up = NR |
| Arnal et al.^35^  (2012)  France | RCT crossover | - n = 50  - Age, mean (SD), *y* = 65 (16)  - Gender, *n* (%) = M: 32 (64%) F: 18 (36%)  - Eligibility = invasively and passively ventilated patients with moderate severity of ARF.  - Exclusion criteria = increased intracranial pressure, severe ARDS requiring a permissive hypercapnia strategy, chronic respiratory failure receiving long-term oxygen therapy and/or home non-invasive ventilation, severe cardiac arrhythmia, therapeutic hypothermia, brain-dead patients, pregnancy and bronchopleural fistula. | - IMV with closed-loop control of oxygen (INTELLiVENT^®^–ASV)  - Duration = 2-hours  - Follow-up = NR | - IMV with manual control of oxygen (ASV mode)  - Duration = 2-hours  - Follow-up = NR |
| Arnal et al.^36^  (2018)  France | RCT | - n = 60  - Age, mean (SD), *y* = Exp 67 (12); Con 62 (16)  - Gender, *n* (%) = Exp M: 20 (66.7%), F: 10 (33.3%); Con M: 22 (73.3%), F: 8 (26.7%)  - Eligibility = MV patients (expected duration ≥ 48 h)  - Exclusion criteria = brain injured patients with GCS < 6, broncho-pleural fistula, chronic or acute dyshemoglobinemia, ventilation drive disorder such as Cheynes Stokes breathing, moribund patient, patient with “Do Not Resuscitate” order before inclusion, chronic respiratory disease requiring long term home ventilation, patient ventilated for planned surgery, pregnancy, lack of informed consent, and the participation in another clinical study. | - IMV with closed-loop control of oxygen (INTELLiVENT^®^–ASV)  - Duration = 4-days  - Follow-up = 4-days | - IMV with manual control of oxygen  - Duration = 4-days  - Follow-up = 4-days |
| Bialais et al.^37^  (2016)  Belgium | RCT | - n = 70  - Age, median [IQR], *y* = Exp 59 [47-69], Con 58 [44-71]  - Gender, n = Exp: M: 25; F: 17; Con M: 22; F: 16  - Eligibility = MV patients (duration ≥ 48 h) and BMI < 40 kg/m².  - Exclusion criteria = ventricular assistance with intra-aortic balloon counterpulsation, bronchopleural fistula, CO_2_ gradient higher than 15 mmHg, pregnancy, adults under guardianship, people deprived of freedom and inclusion in another study protocol. | - IMV with closed-loop control of oxygen (INTELLiVENT^®^–ASV)  - Duration = 48-hours  - Follow-up = NR | - IMV with manual control of oxygen  - Duration = 48-hours  - Follow-up = NR |
| Buiteman-Kruizinga et al.^53^ (2022) Netherlands | RCT  crossover | - n = 18  - Age, *y* = NR  - Gender = NR  - Eligibility = patients within 24-hour of start of ventilation and expected to need MV > 24 hours.  - Exclusion criteria = MV through a tracheostomy cannula, BMI > 40, any contraindication for use of the automated ventilation mode. | - IMV with closed-loop control of oxygen (INTELLiVENT–ASV)  - Duration = 3-hours  - Follow-up = NR | - IMV with manual control of oxygen  - Duration = 3-hours  - Follow-up = NR |
| Chelly et al.^38^  (2020)  France | RCT crossover | - n = 265  - Age, mean (SD), *y* = 64 (14)  - Gender, *n* (%) = M: 172 (65); F: 93 (35)  - Eligibility = MV for at least 48 h, with a FiO_2_ ≤ 60%.  - Exclusion criteria = prone positioning, use of neuromuscular blocking agents, pregnant women, contraindication to automated ventilation mode (delirium, broncho-pleural fistula, respiratory drive disorder such as Cheyne-Stokes breathing), and low-quality measurement for SpO_2_. | - IMV with closed-loop control of oxygen (INTELLiVENT^®^–ASV)  - Duration = 6-hours  - Follow-up = NR | - IMV with manual control of oxygen  - Duration = 6-hours  - Follow-up = NR |
| Clavieras et al.^39^  (2013)  France | RCT crossover | - n = 7  - Age, median [IQR], *y* = 58 [50-64]  - Gender, *n* = M: 10; F: 4  - Eligibility = MV patients (spontaneous mode), with an expected duration of MV > 48 h.  - Exclusion criteria = clinical instability, decision to withhold life-sustaining treatment, pregnant women. | - IMV with closed-loop control of oxygen (INTELLiVENT^®^–ASV)  - Duration = 24-hours  - Follow-up = NR | - IMV with manual control of oxygen  - Duration = 24-hours  - Follow-up = NR |
| De Bie et al.^40^  (2020)  Netherlands | RCT | - n = 220  - Age, median [IQR], *y* = Exp 70 [62-76], Con 70 [63-76]  - Gender, *n* (%) = Exp M: 73 (67), F: 36 (33); Con M: 80 (72.1), F: 31 (27.9)  - Eligibility = elective cardiac surgery requiring IMV in the ICU.  - Exclusion criteria = before surgery: BMI >35 kg m^2^, history of pneumonectomy or lobectomy,  COPD (Global Initiative for Chronic Obstructive Lung Disease Class III or IV), already enrolled in another interventional trial. After cardiac surgery: ECMO, hemodynamic instability, fast-track cardiac surgery. | - IMV with closed-loop control of oxygen (INTELLiVENT^®^–ASV)  - Duration = 3-hours  - Follow-up = 30-day | - IMV with manual control of oxygen  - Duration = 3-hours  - Follow-up = 30-day |
| Denault et al.^41^  (2020)  Canada | RCT crossover | - n = 30  - Age, mean (SD), *y* = 62 (9)  - Gender, *n* (%) = M: 23 (76.7), F: 7 (23.3)  - Eligibility = patients with mild and moderate obesity who underwent CABG as sole procedure.  - Exclusion criteria = COPD, restrictive syndrome other than one associated with obesity, or obstructive sleep apnea requiring CPAP to focus on obesity as a sole potential risk factor for hyperoxia-induced hypercapnia. | - NIV closed-loop control of oxygen (FreeO2 system) with a SpO_2_ target of 90±2% (conservative)  - Duration = 30-min  - Follow-up = NR | - Noninvasive manual control of oxygen with a SpO_2_ target of 95% (liberal)  - Duration = 30-min  - Follow-up = NR |
| Eremenko et al.^42^  (2021)  Russia | RCT | - n = 80  - Age, mean (SD), *y* = Exp 59 (8), Con 59.2 (10.8)  - Gender, *n* (%) = M: 57 (71.2), F: 23 (28.8)  - Eligibility = aged 30-76 years; uncomplicated cardiac surgery; BMI: 18-35 kg/m^2^  - Exclusion criteria = before surgery: severe renal, hepatic or cardiac failure. After surgery: acute myocardial infarction, hemodynamic instability, PaO_2_/FiO_2_ < 150, allergic reaction, seizures, delirium, acute stroke. | - IMV with closed-loop control of oxygen (INTELLiVENT^®^–ASV)  - Duration = total duration of MV support  - Follow-up = hospital length of stay | - IMV with manual control of oxygen  - Duration = total duration of MV support  - Follow-up = hospital length of stay |
| Hansen et al.^43^  (2018)  Denmark | RCT crossover | - n = 19  - Age, mean (SD), *y* = 72.4 (8.4)  - Gender, *n* (%) = M: 12 (60), F: 8 (40)  - Eligibility = COPD exacerbation patients with an estimated LOS > 48 h and SpO_2_ ≤ 88% on room air.  - Exclusion criteria = other significant respiratory or cardiac conditions causing hypoxemia, severe ongoing malignancy, high risk for need of mechanical ventilation. | - Nasal cannula with closed-loop control of oxygen (O2matic system)  - Duration = 4-hours  - Follow-up = NR | - Nasal cannula with manual control of oxygen  - Duration = 4-hours  - Follow-up = NR |
| Harper et al.^44^  (2021)  New Zealand | RCT | - n = 20  - Age, mean (SD), *y* = Exp 68.7 (17), Con 69.2 (19)  - Gender, *n* (%) = Exp M: 6 (60) F: 4 (40), Con M: 6 (60) F: 4 (40)  - Eligibility = patients with HFNC with a prescribed SpO_2_ target ranges from (88-92%) or (92-96%).  - Exclusion criteria = incapability of brief interruption in oxygen or high-flow therapy, domiciliary use of CPAP or NIV, obstructive sleep apnoea, respiratory infection or colonization with multidrug resistant bacteria, infection with SARS-CoV-2, haemodynamic instability, end of life care, risk of barotrauma, as assessed by the investigator, nasal or facial conditions precluding use of HFNC, intracranial trauma or trans-nasal neurosurgery (within 6 weeks), any condition which limits the feasibility of continuous SpO2 monitoring, pregnancy or breastfeeding, cognitive impairment or impaired consciousness precluding informed consent, implanted electronic medical device, any other condition which, at the investigator’s discretion, is believed may present a safety risk or impact the feasibility of the study or the study results. | - HFNC with closed-loop control of oxygen (Airvo3 system)  - Duration = 24-hours  - Follow-up = NR | - HFNC with manual control of oxygen (Airvo2 system)  - Duration = 24-hours  - Follow-up = NR |
| Huynh Ky et al.^50^  (2017)  Canada | RCT | - n = 60  - Age, mean (SD), *y* = 63 (12)  - Gender, *n* = M: 44, F: 16  - Eligibility = patients with acute coronary syndrome.  - Exclusion criteria = severe COPD diagnosis. | - Exp 1: NIV closed-loop control of oxygen (FreeO_2_ system) with 92% of SpO_2_ target  - Exp 2: NIV closed-loop control of oxygen (FreeO2 system) with 96% of SpO_2_ target  - Duration = 24-hours  - Follow-up = NR | - Noninvasive manual control of oxygen (FreeO_2_ system)  - Duration = 24-hours  - Follow-up = NR |
| Johannigman et al.^45^  (2009)  USA | RCT crossover | - n = 15  - Age, mean (SD), *y* = 35.6 (13.2)  - Gender, *n* (%) = M: 13 (86.7), F: 2 (13.3)  - Eligibility = trauma patients requiring IMV; current FiO_2_ >35%; indwelling arterial line; and ability to monitor SpO_2_ accurately  - Exclusion criteria = NR | - IMV with closed-loop control of oxygen (Impact 754, Eagle)  - Duration = 4-hours  - Follow-up = 8-hours | - IMV with manual control of oxygen (Impact 754, Eagle)  - Duration = 4-hours  - Follow-up = 8-hours |
| Kobayashi et al.^51^  (2017)  Japan | RCT | - n = 48  - Age, y = NR  - Gender = NR  - Eligibility = patients undergoing cardiac surgery.  - Exclusion criteria = NR | - IMV with closed-loop control of oxygen (INTELLiVENT^®^–ASV)  - Duration = 6-hours  - Follow-up = NR | - IMV with manual control of oxygen (ASV mode)  - Duration = 6-hours  - Follow-up = NR |
| Komnov et al.^54^ (2023)  Russian | RCT | - n= 32 - Age, y = NR  - Gender = NR  - Eligibility: Adult patients (age >30 years), elective cardiac surgery, BMI > 35 kg/m^2^ and postoperative treatment with MV  - Exclusion criteria = NR | - IMV with closed-loop control of oxygen (INTELLiVENT^®^–ASV)  - Duration = total duration of MV support  - Follow-up = hospital LOS | - IMV with manual control of oxygen  - Duration = total duration of MV support  - Follow-up = hospital LOS |
| L’Her et al.^46^  (2017)  France | RCT | - n = 187  - Age, mean (SD), *y* = Exp 74.6 (13.2), Con 77.7 (12.2)  - Gender, *n* (%) = Exp M: 56 (60.9), F: 37 (39.1); Con M: 48 (51.1), F: 46 (48.9)  - Eligibility = emergency department admission, within < 2 h for acute respiratory disorder requiring ≥ 3 L/min of O_2_ (SpO_2_ target ≥ 92%).  - Exclusion criteria = life-threatening hypoxaemia, clinical signs of ventilatory assistance requirement, emergent surgery or coronary angiography, pregnant or breastfeeding women, patients under administrative protective measures. | - Mask to administer either low or high O_2_ flow with closed-loop control of oxygen (FreeO2 system)  - Duration = 3-hours  - Follow-up = hospital length of stay | - Mask to administer either low or high O_2_ flow with manual control of oxygen  - Duration = 3-hours  - Follow-up = hospital length of stay |
| L’Her et al.^47^  (2021)  France | RCT | - n = 198  - Age, mean (SD), *y* = Exp 62.2 (12.6), Con 63.1 (13.5)  - Gender, (%) = Exp M: 55.2, F: 44.8; Con M: 55.3, F: 44.7  - Eligibility = patients undergoing abdominal or thoracic surgery; ARISCAT ≥ 26; indication of ventilatory support after surgery.  - Exclusion criteria = BMI ≥ 35; obstructive sleep apnea and pregnant patients. | - Nasal cannula/prongs (low flow) or open face mask (low or medium flow) with closed-loop control of oxygen (FreeO2 system)  - Duration = 3-hours/3-days  - Follow-up = 28-days | - Nasal cannula/prongs (low flow) or open face mask (low or medium flow) with manual control of oxygen  - Duration = 3-hours/3-days  - Follow-up = 28-days |
| Lellouche et al.^52^  (2013)  Canada | RCT | - n = 60  - Age, mean (SD), *y* = Exp 67.7 (8.8), Con 65.3 (9.7)  - Gender, *n* (%) = Exp M: 23 (76.7), F: 7 (23.3); Con M: 22 (73.3), F: 8 (26.7)  - Eligibility = pre inclusion criteria were: (1) elective cardiac surgery; (2) age 18-90 years; (3) BMI < 40 kg/m^2^; (4) baseline PaCO_2_ < 50 mmHg; and (5) serum creatinine < 200 µmol/L. After surgery, hemodynamically stable, and urine output > 50 mL/h.  - Exclusion criteria = bronchopleural fistula, study ventilator not available. | - IMV with closed-loop control of oxygen (INTELLiVENT^®^–ASV)  - Duration = 4-hours  - Follow-up = ICU length of stay | - IMV with manual control of oxygen  - Duration = 4-hours  - Follow-up = ICU length of stay |
| Lellouche et al.^48^  (2016)  Canada | RCT | - n = 50  - Age, mean (SD), *y* = Exp 71 (8), Con 73 (8)  - Gender, *n* (%) = Exp M: 15 (60), F: 10 (40); Con M: 12 (48), F: 13 (52)  - Eligibility = hospitalized COPD exacerbation patients with oxygen therapy prescription; age ≥ 40 years; and past or current smoking history of at least 10 pack-years.  - Exclusion criteria = admitted for > 24 hours, multidrug-resistant bacteria infection, intermittent noninvasive ventilation, cognitive impairment. | - Nasal cannula or nonocclusive mask with closed-loop control of oxygen (FreeO2 system)  - Duration = 7-days  - Follow up = 180-days | - Nasal cannula or nonocclusive mask with manual control of oxygen  - Duration = 7-days  - Follow up = 180-days |
| Roca et al.^49^  (2022)  pain | RCT crossover | - n = 45  - Age, mean (SD), *y* = 35.6 (13.2)  - Gender, *n* (%) = M: 24 (53.3), F: 21 (46.7)  - Eligibility = ICU admission receiving HFNC with flow rate ≥ 40 L/min with FiO_2_ ≥ 30%, and expected to receive HFNC for at least 8 hours after randomization.  - Exclusion criteria = pregnant women, indication or high risk for immediate intubation, indication for non-invasive ventilation, haemodynamic instability, severe acidosis, poor SpO_2_ signal, chronic or acute dyshaemoglobinaemia, tracheostomy, previously enrolled in this trial or enrolled in another interventional study could not participate. | - HFNC with closed-loop control of oxygen  - Duration = 4-hours  - Follow-up = NR | - HFNC with manual control of oxygen  - Duration = 4-hours  - Follow-up = NR |

3. Hyperoxemia is equally detrimental especially in patients with Type 2 respiratory failure patients. What were the causes of hyperoxemia in closed-loop systems should be briefly discussed.

**Thank you for your comment and question. The percentage of time with hyperoxemia ranged from less than 1% to 35% in the closed-loop group and less than 1% to 31% in the manual titration group, and this was found to be not statistically different. Of note, in most of the studies, the duration of hyperoxemia was short. In the one study with a high percentage of time with hyperoxemia (Arnal, 2018), utilization of INTELLiVENT–ASV resulted in 35% of time with hyperoxemia versus 31.33% with manual titration. We added the following to the revised version of the manuscript:**

- **[Page 13; line 15] *‘However, it may not effectively reduce the occurrence of hyperoxemia, particularly in mechanical ventilation systems, or when high oxygen targets are being used. For instance, one trial [36] showed a high incidence of hyperoxemia with automatic oxygen control; however, this incidence was also high in the control group (35% vs. 33%).’***

4. Subgroup analysis based on duration of intervention should be done as there are various studies having intervention for more than 24 hours.

**Thank you for this valuable suggestion. See also our response to your comment 5, below. According to this suggestion, and also your suggestion in comment 5, we conducted subgroup analyses considering the duration of the intervention, and type of ICU admission, focusing on the primary endpoint of our study. We added the findings as a post hoc analysis to the supplement, and added the following to the revised version of the manuscript:**

- **[Page 8; line 21] *‘In addition, we conducted two posthoc analyses, one comparing the percentage of time in the SpO*_2_ *target for subgroups according to duration of the intervention, and one in subgroups according to the reason for admission, i.e., medical or surgical.’***
- **[Page 10; line 11] *‘In 7 studies [37,40,44,46–49], closed-loop oxygen titration devices increased the percentage of time in the predefined SpO*_2_ *target with substantial heterogeneity, not completely explained by the planned and post hoc subgroup analysis (Fig 4, S2 Fig, S3 Fig and S11 Table).’***
- **[Supporting information page 5] *‘S2 Fig. Forest plot of subgroup analysis of duration of intervention for the percentage of time spent in the SpO*_2_ *target.’***
- **[Supporting information page 6] ‘S3 Fig. Forest plot of subgroup analysis of patient’s condition (medical or post-surgical) for the percentage of time spent in the SpO_2_.'**

5. Separate analysis for studies with post-surgical patients and patients with medical conditions requiring supplemental oxygen should be considered.

**See our reply to your comment 4, above.**

**Reviewer 2**

1. The aim of your study was to evaluate whether closed-loop oxygen control systems are effective, efficient and safe. Despite heterogeneity of studies it was seen that Closed-loop oxygen titration are effective in reducing costs, oxygen consumption and healthcare workload. Are they safe - No; as they resulted in longer patient times with hyperoxemia. Are they efficient - No; as time for weaning from supplemental oxygen's not shown to reduce with these systems and they had no effects on ventilator associated adverse events, length of stay, and mortality. Please include the same clearly in conclusions.

**Thank you for your comments and suggestions. We agree that there remains a substantial gap in our understanding of the safety and efficacy of closed-loop oxygen systems in reducing mortality rates and shortening hospital stays.**

**We revised the conclusion to better address these concerns raised, as follows:**

- ***[Page 17; line 6]‘In summary, while closed-loop oxygen titration systems probably increase the percentage of time spent within desired SpO_2_ ranges and reduce healthcare workload and costs, their safety remains uncertain. Adverse events are likely underreported in existing studies. Moreover, the extent to which these systems enhance efficiency, such as by reducing the duration of oxygen weaning, and even more importantly, by affecting length of stay and mortality rates, remains unclear. This uncertainty primarily stems from the limited duration of the intervention in the scarce, and small existing studies. Future research should focus on evaluating effectiveness, safety, and efficacy over longer periods, and prioritize patient-centered endpoints.’***

**Reviewer 3**

1. This meta-analysis does not have a funnel plot to look at the publication bias. There is considerable heterogeneity (I^2^=80.1%) and lacks a sensitivity analysis.

**Thank you for your comment regarding the 'funnel plot' and sensitivity analysis. We appreciate and agree with your suggestion. We have incorporated sensitivity analysis for the main outcomes, as follows:**

- **[Page 10; line 14] *‘The sensitivity analysis for the percentage of time in the SpO2 target, included only trials with a low risk of bias (S4 Fig.) and showed similar estimates of the intervention and inconsistency. There's no clinically important difference in effect estimates between the meta-analysis encompassing all studies and the one focusing solely on studies with a low risk of bias, any inconsistency is likely to be insignificant [55].’***

[Supporting information page 7] ***S4 Fig.*** *Sensitivity analyses for the percentage of time spent in the SpO_2_ target*

[Page 12; line 15] *‘In the sensitivity analyses excluding trials with some concerns according to the risk of bias assessment (S8 Fig), we observed a slight increase in the heterogeneity (I² = 83%) maintaining the direction of the effect of interventions (MD -4.94, 95% CI -9.43 to -0.46).’*

[Supporting information page 13] ***S8 Fig.*** *Sensitivity analyses for healthcare professional’s workload*

**We fully agree that the absence of a Funnel Plot analysis is a limitation of our systematic review. Despite the inclusion of only seven studies in the meta-analysis, we also performed the funnel plot for the percentage of time in the SpO2 target (see the figure below). However, the test for publication bias is severely underpowered for this outcome, and visual inspection of a funnel plot with few studies can be highly misleading. Thus, this result should be interpreted with caution. We have incorporated this information into the results and discussion section, as follows:**

- **[Page 10; line 19] *‘Despite the observed asymmetry in the funnel plot (S5 Fig.), we cannot definitively attribute it to publication bias due to the limited number of studies included in this outcome.’***
- **[Page 16; line 16] *‘Second, despite our considerable efforts to mitigate publication bias, we were unable to reliably assess it through the funnel plot due to the limited number of available studies.’***

[Supporting information page 8] ***S5. Fig.*** *Funnel plot for the percentage of time spent in the SpO2 target.*

**Your advice aligns with the suggestions made by other reviewers above, where subgroup analyses have been proposed with the aim of exploring how effect varies across different groups. (please refer to our response to comments 4 and 5 by reviewer '1').**
